# Oral Microbiome in Systemic Autoimmune Diseases: A Systematic Review

**DOI:** 10.1111/odi.70215

**Published:** 2026-03-27

**Authors:** Sophie Jung, Eirini Militsi, Olivier Huck

**Affiliations:** ^1^ Centre de Référence Maladies Rares Orales et Dentaires (O‐Rares), Pôle de Médecine et de Chirurgie Bucco‐Dentaires Hôpitaux Universitaires de Strasbourg Strasbourg France; ^2^ Faculté de Chirurgie Dentaire Université de Strasbourg Strasbourg France; ^3^ INSERM UMR_S 1109 “Molecular ImmunoRheumatology” Université de Strasbourg Strasbourg France; ^4^ Unité Fonctionnelle de Parodontologie, Pôle de Médecine et de Chirurgie Bucco‐Dentaires Hôpitaux Universitaires de Strasbourg Strasbourg France; ^5^ INSERM UMR_S 1260 “Regenerative NanoMedicine” Université de Strasbourg Strasbourg France

**Keywords:** autoimmune disease, microbiome, oral, periodontal

## Abstract

**Objective:**

The oral cavity represents a key but underexplored interface between host immunity and microbial communities. The aim of this systematic review was to synthesize current literature on oral microbiota alterations in systemic autoimmune diseases.

**Methods:**

PubMed and Web of Science databases were searched for human studies published between January 2000 and April 2025. Eligible observational studies compared adults with diagnoses of systemic autoimmune diseases to controls and characterized oral microbiota diversity and/or composition using sequencing‐based methods. Different oral habitats were analyzed (saliva, dental plaque, oral mucosa, gingival crevicular fluid).

**Results:**

42 studies met inclusion criteria: 19 on rheumatoid arthritis, 18 on primary Sjögren's syndrome, 5 on systemic lupus erythematosus, and 1 on anti‐neutrophil cytoplasmic autoantibody‐associated vasculitis. 16S rRNA gene sequencing predominated and only 3 studies used shotgun metagenomics, among which one also profiled the oral virome. Across systemic autoimmune diseases, dysbiosis was characterized by enrichment of anaerobic genera (*Prevotella, Veillonella*) and depletion of commensals (*Neisseria, Haemophilus*), with distinct β‐diversity separation from controls. Periodontal disease and reduced salivary secretion significantly modulated microbial communities but did not fully explain disease‐associated alterations.

**Conclusion:**

The oral microbiome exhibited shared dysbiotic signatures. However, methodological and clinical heterogeneity limited direct comparison between studies.

## 1 Introduction

Systemic autoimmune diseases (AID) are multifactorial disorders characterized by a breakdown of immune tolerance, leading to progressive tissue and organ damage (Davidson and Diamond 2001). The most prevalent AID include rheumatoid arthritis (RA), systemic lupus erythematosus (SLE), and primary Sjögren's syndrome (pSS), but rarer entities such as systemic sclerosis (SSc), primary antiphospholipid syndrome (APL), mixed connective tissue disease (MCTD), myositis, and systemic vasculitis (e.g., anti‐neutrophil cytoplasmic autoantibody [ANCA]‐associated vasculitis [AAV]) also belong to this spectrum. Their heterogeneous and often overlapping clinical manifestations make diagnosis and management challenging (Fugger et al. 2020; Miller 2023).

Systemic AID arise from a complex interplay between genetic susceptibility and environmental triggers (Pisetsky 2023). Among environmental factors, dysbiosis has been increasingly implicated in the multistep pathogenesis of autoimmunity (Belkaid and Hand 2014; Levy et al. 2017; Pereira and Kriegel 2023). The oral cavity hosts one of the most diverse microbial ecosystems, comprising more than 700 bacterial species (Dewhirst et al. 2010), as well as fungi, viruses, and protozoa, organized into distinct ecological habitats including saliva, dental plaque, mucosa, and gingival crevicular fluid (GCF). Periodontal biofilms form highly structured site‐specific communities, especially within subgingival pockets that provide anaerobic conditions favorable to pathogens such as *Porphyromonas gingivalis* (*Pg*) (Wade 2013).

Compared with the gut, the oral microbiota has been relatively underexplored in systemic autoimmunity (Peng et al. 2022; Suárez et al. 2020). Oral dysbiosis can sustain chronic local inflammation and barrier disruption, but it may also promote systemic immune activation through persistent low‐grade inflammation (Greiling et al. 2018; Hajishengallis and Chavakis 2021; Spivak et al. 2022; Suárez et al. 2020; Vieira et al. 2018). RA was the first systemic AID to highlight a potential causal role for oral dysbiosis in systemic immune dysregulation. *Pg* (via its peptidylarginine deiminase [PPAD]) and, more recently, *Aggregatibacter actinomycetemcomitans* (*Aa*), have been mechanistically linked to RA through their capacity to promote host protein citrullination, potentially inducing anti‐citrullinated protein antibodies (ACPA), a serological hallmark of the disease (Culshaw et al. 2011; Konig et al. 2016; Petit et al. 2024; Wegner et al. 2010).

Microbiome studies mainly rely on 16S rRNA gene sequencing, which provides bacterial taxonomic profiling, but no direct functional information. Shotgun metagenomic sequencing enables species‐ and strain‐level identification as well as functional pathway analysis from total microbial DNA, but with greater cost and analytical complexity.

The aim of this systematic review was to synthesize current evidence on the oral microbiota in systemic AID, identify disease‐specific and shared microbial signatures, and discuss potential biological pathways linking oral dysbiosis to systemic immune activation.

## 2 Methods

### 2.1 Search Strategy

**FIGURE 1:**
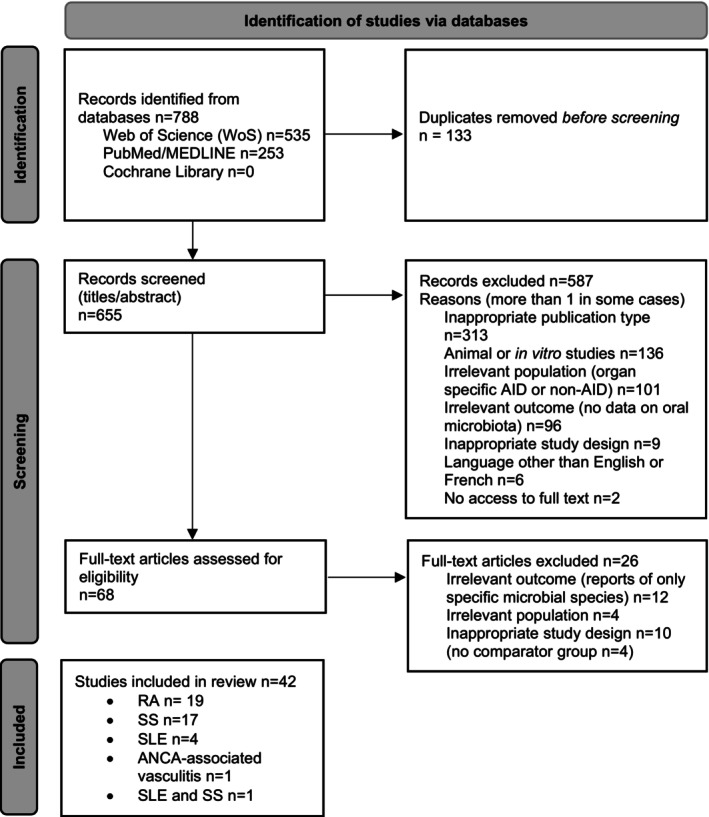
PRISMA flowchart. AID, autoimmune disease; ANCA, anti‐neutrophil cytoplasmic antibody; pSS, primary Sjögren's syndrome; RA, rheumatoid arthritis; SLE, systemic lupus erythematosus.

PRISMA 2020 guideline (Page et al. 2021) (Figure 1 and Table S1) were followed and protocol was registered on Open Science Framework (https://doi.org/10.17605/OSF.IO/W27YU). PubMed/MEDLINE and Web of Science (WoS) online databases were searched independently by S.J. and O.H. (January 2000–April 2025) for human observational studies.

### 2.2 Eligibility Criteria

According to PECO framework (Morgan et al. 2018), eligible studies compared adults with validated systemic AID diagnoses to controls and characterized oral microbiota (saliva, dental plaque, mucosa, and/or GCF) composition and/or diversity using sequencing.

### 2.3 Data Collection

Data extracted included study design, sampling sites, oral status, sequencing methods, and key microbial findings.

Full search equations, eligibility criteria, systemic AID classification criteria, and selection process are described in Methods S1.

## 3 Results

### 3.1 Study Selection Process

A total of 788 records were identified. After removing 133 duplicates and screening 655 records, 42 studies were included in the qualitative synthesis: 19 on RA, 18 on SS, 5 on SLE, and 1 on AAV, with one study addressing both SS and SLE (Figure 1). Most used 16S rRNA gene sequencing (39/42 studies), and three employed shotgun metagenomics (Cheng et al. 2021; Guo et al. 2022; Zhang et al. 2015).

### 3.2 Rheumatoid Arthritis (Ra)

#### 3.2.1 Study Characteristics

Nineteen case–control studies (Table 1) profiled RA cohorts spanning Asia, Europe and the Americas (*n* = 1279 patients), predominantly women. Most studies compared RA patients to healthy controls (HCs, *n* ≈ 900). Several studies also included at‐risk individuals, i.e., individuals with clinically suspect arthralgia (Arleevskaya et al. 2022) or ACPA/rheumatoid factor (RF)‐positive but not yet diagnosed with RA (Cheng et al. 2021; Kroese et al. 2021; Tong et al. 2019), or patients with osteoarthritis (OA) as disease controls (Chen et al. 2018; Mikuls et al. 2018). (Table 1). Sixteen studies used 16S rRNA gene sequencing and three Shotgun metagenomics (Cheng et al. 2021; Guo et al. 2022; Zhang et al. 2015), among which one also profiled the virome (Guo et al. 2022) (Table 2). Subgingival plaque was most sampled (*n* = 13), followed by saliva (*n* = 6) and swabs (*n* = 6). Four studies sampled multiple sites (Guo et al. 2022; Kozhakhmetov et al. 2023; Kroese et al. 2021; Zhang et al. 2015) (Table 1).

**TABLE 1:** Characteristics of the study populations.

AID	References	Sample size	Oral sample types	Matching, confounding factors and exclusion criteria	Oral status
RA	Arleevskaya et al. (2022)	15 early RA patients 47 pre‐RA patients with clinically suspect arthralgia (CSA) 22 HCs (CSA = 0) Only ♀	Buccal swabs	Only ♀ Data on behavior factors (smoking, alcohol consumption, overweight status, etc.)	—
	Corrêa et al. (2019)	42 RA patients (18♀): 21 with PD (50%) and 21 without PD 47 HCs (20♀): 20 with PD (43%) and 27 without PD	Subgingival dental plaque	Age‐ and sex‐matched	Number of missing teeth; periodontal parameters: PI, PPD, CAL, BoP; oral hygiene habits ↑PD and CAL in RA patients with PD vs HCs with PD
	Cheah et al. (2022)	49 RA patients (40♀): 22 with PD, 8 with G, 19 PH 51 HCs = non‐RA (31♀): 27 with PD, 9 with G, 15 PH	Subgingival dental plaque	Average age different between RA and HCs (RA > HCs) PD severity matched between RA‐PD and non‐RA PD Exclusion criteria: non‐Malaysian, AB, periodontal treatment < 4 months, other systemic disease and AID, pregnancy Smoking status known	Number of teeth; periodontal parameters: PI, GI, CAL, PPD, PISA; oral hygiene habits
	Chen et al. (2018)	110 RA patients (90 ♀) 67 OA patients (46 ♀) 155 HCs (75 ♀)	Unstimulated saliva	Not sex‐ and age‐matched (RA and OA older than HCs, more ♀ in RA) Exclusion criteria: apparent oral disease including periodontal disease	No data except that individuals have no apparent oral disease
	Chen et al. (2022)	30 RA patients (25 ♀): 21 with PD, 19 PH 25 HCs (19 ♀): 17 with PD, 8 PH 3 groups after matching (sex‐, age‐ and diabetes status‐matching): “All matched” (AM) group (no matching on the periodontal status): 21 RA and 21 HCs PD: 12 RA and 12 HCs PH: 6 RA and 6 HCs	Subgingival dental plaque	Sex‐, age‐ and diabetes status‐matching Exclusion criteria: smoking, malignancy, pregnancy, AB Data on behavior factors	Periodontal parameters: PPD, CAL, BoP; X‐rays
	Cheng et al. (2021)	48 CCP^+^ at‐risk patients (musculoskeletal symptoms but no synovitis) 26 early RA patients 32 HCs	Subgingival dental plaque (3 PH sites +3 PD sites)	Balanced for sex, age, smoking status	Periodontal parameters: PPD, CAL, BoP
	de Jesus et al. (2021)	35 RA patients (29 ♀) 64 non‐RA controls (32 ♀; 58 first‐degree relatives at‐risk for future RA)	Buccal swabs	Not sex‐ and age‐matched (RA older than non‐RA, more ♀ in RA) Data on individual behavior factors (smoking, BMI, etc.): ns between RA and non‐RA	Self‐administered oral health questionnaire (denture, gum bleeding, feeling of teeth mobility, oral hygiene habits, etc.)
	Eriksson et al. (2022)	53 RA patients with PD (46 ♀) 48 non‐RA patients with PD (26 ♀)	Stimulated saliva	Similar age Not sex‐matched Exclusion criteria: other forms of arthritis, pregnancy, no recent periodontal treatment, AB	PD diagnosis based on periodontal parameters measurement: PPD, CAL, BoP Severe PD: 8% of RA and 15% of non‐RA (ns difference)
	Kozhakhmetov et al. (2023)	75 RA patients: 16 with PD, 59 PH 114 HCs: 20 with PD, 94 PH Only ♀	Tongue, gums and tonsils scrapings Dental plaque	Similar age Smoking status known Exclusion: pregnancy, cancer, other systemic disease, AB and probiotics	Oral health questionnaire (bad breath, gum bleeding, feeling of teeth mobility, etc.) Periodontal parameters: PPD, CAL, BoP, X‐rays Severe PD: 21.3% of RA and 17.5% of HCs (ns difference)
	Kroese et al. (2021)	50 early RA patients (39♀) 69 at‐risk RA (arthralgia and ACPA/RF+; 38♀) 50 HCs (39♀)	Subgingival dental plaque Tongue swabs Stimulated saliva	Age‐ and sex‐matched Data on several individual cofounding factors (smoking, alcohol consumption, AB, drugs, etc.)	Number of teeth; DMFT; periodontal parameters: CPITN, BoP, PPD, PISA; oral hygiene habits Periodontal parameters, PD prevalence, DMFT: no significant difference between the groups
	Lehenaff et al. (2021)	8 RA patients (5 ♀; under DMARDs but no biologic agents): 1 PH, 3 with PD stage I, 1 with PD stage II, 3 with PD stage III 10 non‐RA controls (5 ♀; same family): 4 with PD stage I, 4 with PD stage II, 2 with PD stage III	Subgingival dental plaque (1 PH site +1 deep subgingival PD‐associated site)	Similar age and sex repartition Smoking status and BMI known Exclusion criteria: < 8 teeth, AB, immune compromising conditions other than RA	Number of caries PD diagnosis based on periodontal parameters measurement: PPD, CAL, BoP No significant difference in number of caries and periodontal health status between RA and non‐RA
	Liu et al. (2020)	54 RA patients 45 patients with PD 44 HCs	Subgingival dental plaque	No matching (random) Average age different between groups (RA > PD and HCs) No data on gender, exclusion criteria	No data on periodontal parameters PD patients recruited according to clinical criteria from 1992 (Machtei et al. 1992)
	Liu et al. (2024)	47 RA patients (40 ♀): 34 in active phase (according to DAS28; 29 ♀), 13 in inactive phase (11 ♀) 10 HCs (8 ♀)	Dorsal tongue swabs	No matching Similar gender Average age different between groups (RA>HCs) No data on exclusion criteria Smoking status known	—
	Lopez‐Oliva et al. (2018)	22 RA patients (17 ♀) 19 HCs (13 ♀) All PH	Subgingival dental plaque	No matching Smoking status and alcohol consumption known Average age different between RA patients and HCs (RA > HCs) Exclusion criteria: other rheumatic disease, recent surgical procedure, AB	Periodontal parameters: PPD, BoP, CAL PPD and CAL: statistically significant (but clinically inconsequential) difference between RA and HCs
	Mikuls et al. (2018)	260 RA patients (91 ♀): 99 with PD (38%), 161 without PD 296 OA patients (115 ♀): 81 with PD (27%), 215 without PD	Subgingival dental plaque	Similar demographics (age, gender, ethnic origin) Statistically significant differences in BMI (RA < OA), smoking (RA > OA) and diabetes (RA < OA) status	Evaluation of periodontal parameters but not presented in the paper PD: 38% of RA and 27% of OA (*p* = 0.007)
	Scher et al. (2012)	31 new‐onset RA patients (NORA; disease duration > 6 weeks and absence of DMARDs or steroid treatment): 21 ♀ 34 chronic established RA (CRA; disease duration > 6 months): 27 ♀ 18 HCs: 12♀	Subgingival dental plaque (6 most periodontally diseased sites)	Age‐, sex‐ and ethnicity‐matching for HCs Exclusion criteria: AB, probiotics, current extreme diet (IBD), cancer, gastro‐intestinal tract surgery leaving permanent residua (e.g., gastrectomy, bariatric surgery, colectomy), significant liver, renal or peptic ulcer disease, history of inflammatory arthritis (for HCs)	Periodontal parameters: PPD, BoP, CAL PH: NORA 0%, CRA 6%, HCs: 45% Gingivitis: NORA 13%, CRA 3%, HCs 11% Slight‐moderate PD: 26%, CRA 38%, HCs 23% Severe PD: NORA 62%, CRA 53%, HCs 22% (NRA and CRA vs HCs: *p* < 0.01)
	Tong et al. (2019)	27 RA patients (16 ♀) 29 ACPA^+^ at‐risk patients (±arthritis; 12 ♀) 23 HCs (13 ♀)	Unstimulated saliva	Age‐, sex‐ and ethnicity‐matching for HCs Smoking status known Similar age, gender and smoking status between the groups Exclusion criteria: AB, recent surgery, extreme diet, cancer, organ dysfunction, other rheumatic or AID, treatment with biologic agents	Self‐reported questionnaire for PD assessment (bleeding on brushing, non‐traumatic loose or missing teeth, PD diagnosed by a dentist) PD: 63% RA, 62% at‐risk RA, 57% HCs No significant difference between the groups
	Zhang et al. (2015)	*Dental plaque* 54 treatment‐naïve RA patients (42 ♀) 51 HCs *Saliva* 51 treatment‐naïve RA patients (41 ♀) 47 HCs 43 RA patients with both dental plaque and saliva samples (34 ♀)	Dental plaque Saliva Swabs from posterior pharynx	No data on matching, BMI known Exclusion criteria: AB, infection, cancer, other AID, pregnancy, normal values on recent screens (blood tests and blood pressure) for HCs	—
	Guo et al. (2022)	104 untreated RA patients (new onset) 60 treated RA patients 102 HCs	Saliva (122 samples: 51 untreated RA, 24 treated RA, 47 HCs) Subgingival dental plaque (143 samples: 54 untreated RA, 38 treated RA, 51 HCs)	Age‐, sex‐ and BMI‐matched	—
SS	Alam et al. (2020)	25 pSS patients: 8 without dryness and 17 sicca 11 non‐pSS sicca (drug‐induced: *n* = 9) 25 HCs (without dryness) Only ♀	Oral washings	No smoking, AB, steroid, immunosuppressants Only ♀	—
	de Paiva et al. (2016)	10 SS patients (10♀) 11 HCs (7♀)	Dorsal tongue swabs	SS patients older than HCs	—
	Kim et al. (2022)	23 pSS sicca patients 9 non‐SS sicca controls (drug‐induced: *n* = 6) Only ♀	Saliva from parotid gland (lavage)	Smoking status known (*n* = 1 in SS patients)	Dental loss in *n* = 1 SS patient Measurement of salivary flow rate No detailed assessment performed
	Li et al. (2016)	10 pSS patients 10 HCs Only ♀	Buccal swabs	Matching according to age, teeth number, periodontal and mucosal status Inclusion criteria: treatment with stable dosage of HC ±prednisone for at least 3 months Exclusion criteria: smoking, oral mucosa infection, saliva stimulating agents, fluorides, AB, antifungals and previous periodontal treatment, patients with removable dentures and/or implants	Clinical evaluation of oral mucosa and teeth number Measurement of UWS/SWS flow rates (↓ in pSS patients)
	Martínez‐Nava et al. (2023)	48 pSS patients (45♀) 17 HCs (16♀)	GCF	Age‐ and sex‐matched No AB, active infection	For pSS patients: periodontal status (92% with PD; 52% with moderate/severe PD), number of missing, filled and decayed teeth, measurement of UWS flow rate
	Rusthen et al. (2019)	15 pSS patients 15 non‐SS sicca controls 15 HCs Only♀	Stimulated saliva	Similar age, gender, smoking and dental status	Number of missing, decayed, mobile teeth, gingivitis Evaluation of clinical oral dryness score (CODS) and Shortened Xerostomia Inventory (SXI) Measurement of UWS/SWS flow rates
	Saúco et al. (2023)	25 SS patients 25 HCs Only ♀aged ≥ 40 years	Stimulated saliva	Age‐ and sex‐matched No AB, antifungals, antiseptic mouthwashes	Periodontal, mucosal and dental status (DMFT), treatment need Measurement of UWS/SWS flow rates (↓ in 79% SS patients vs 52% HC)
	Sembler‐Møller et al. (2019)	24 pSS patients (22 ♀) 34 non‐SS sicca (30 ♀)	Stimulated saliva	Similar age, gender, smoking, health, dental and periodontal status, UWS/SWS flow rates, intake of xerogenic medications No AB, exclusion of secondary SS	DMFT/S, periodontal status Measurement of UWS/SWS flow rates, oral mucosa examination, palpation of salivary glands and LN Xerostomia more prevalent in non‐SS group
	Sharma et al. (2020)	37 pSS patients 35 HCs Only ♀	Unstimulated saliva	Exclusion: AB, extensive caries, oral ulcerations or candidiasis, dentures, dental procedure within 3 months, diabetes, IBD, renal/liver disease, secondary SS, use of secretagogues, intoxicants	—
	Siddiqui et al. (2016)	9 pSS patients (8 ♀) with normal UWS flow rates 9 HCs (9 ♀)	Unstimulated saliva	No data on matching and exclusion criteria	No data on dental status Normal UWS flow rates
	Tseng et al. (2021)	8 pSS patients 16 HCs Only ♀	Unstimulated saliva	Similar age Exclusion criteria: smoking, other AID, cancer, liver cirrhosis, chronic kidney disease, AB/mouth wash/steroids/immunomodulators within 3 months	—
	van der Meulen et al. (2018a)	37 pSS patients (31♀) 86 non‐SS sicca (69♀) 24 HCs (16♀)	Buccal swabs	Sex‐ but not age‐matched Similar age between pSS and non‐SS sicca but HCs are younger Smocking status known	% of natural teeth Measurement of UWS/SWS flow rates (↓ in pSS and non‐SS sicca vs HCs)
	van der Meulen et al. (2018b)	36 pSS patients (31♀) 85 non‐SS patients (not fulfilling 2016 ACR/EULAR criteria; 67 ♀) 14 HCs (13 ♀)	Oral washings	No matching Smoking status known (20% of non‐SS)	% of natural teeth Measurement of UWS/SWS flow rates (↓ in pSS and non‐SS vs HCs)
	van der Meulen et al. (2019)	For oral samples: 34 SLE patients (28 ♀), 34 pSS patients, no HC	Oral washings Buccal swabs	*SLE vs pSS*: Similar sex, BMI and smoking status	Number of teeth (complete or edentulous) and self‐rated oral health including dry mouth Less severe xerostomia in SLE vs pSS patients
	Wang et al. (2022)	For oral samples: 133 pSS patients (118 ♀) 56 non‐SS patients (49 ♀) 40 HCs (33 ♀),	Oral washings	Similar age and sex repartition Exclusion criteria: other AID, infections, cancer, urogenital or metabolic diseases, AB	—
	Xie et al. (2024)	35 pSS patients (29 ♀) 20 HCs (20 ♀)	Unstimulated saliva Supragingival plaque (only for pSS patients)	Average age different between pSS patients and HCs Exclusion criteria: AB, pregnancy, secondary SS	Oral and dental status not reported Dry mouth: 100% pSS patients
	Zhou, Ling, et al. (2018)	22 pSS patients 23 HCs (no xerostomia) Only ♀	Oral washings	Sex‐ and age‐matched Exclusion criteria: diabetes, radiotherapy, xerogenic medications, oral mucosal lesions	DMFT (significantly ↑ in pSS vs HCs) No xerostomia symptoms in HCs
	Zhou, Cai, et al. (2018)	9 pSS patients (new diagnosis) 5 HCs Only ♀	Unstimulated saliva	Similar age Exclusion criteria: current treatment, AB, other systemic diseases, dental and PD	—
SLE	Corrêa et al. (2017)	52 SLE patients (25 ♀, 35 PD) 52 HCs (22 ♀, 28 PD)	Subgingival dental plaque	Age‐ and sex‐matching No AB	Periodontal status ↑PD prevalence and severity in SLE patients
	Li et al. (2020)	20 SLE patients and 19 HCs	Buccal swabs	Similar age, BMI, and serum levels of nutritional elements No AB and probiotics	—
	Liu et al. (2021)	35 SLE patients (32♀) and 35 HCs (32♀)	Saliva	Age‐ and sex‐matching No antibiotics, probiotics, vitamin D and B12, calcium, oral, contraceptive, metformin, PPI, immunosuppressants	No oral disease or ulceration at inclusion Dental status not reported
	Guo et al. (2023)	100 SLE (94 ♀) patients and 200 HCs (184 ♀) Post treatment cohort: 73 post‐treatment SLE and 146 HCs	Tongue swabs	Age‐, sex‐ and BMI‐matching No antibiotics, probiotics, fermented food	No oral mucosa or gingival diseases Dental status not reported
	van der Meulen et al. (2019)	For oral samples: 34 SLE patients (28 ♀), 34 pSS patients, no HC	Oral washings Buccal swabs	*SLE* vs. *pSS*: Similar sex, BMI and smoking status	Number of teeth (complete or edentulous) and self‐rated oral health including dry mouth Less severe xerostomia in SLE vs. pSS patients
AAV	Esberg et al. (2022)	25 acute AAV patients (15 ♀, 14 with long‐term AB) 23 HCs (12 ♀)	Whole stimulated saliva	Age‐ and sex‐matched 56% AAV patient treated by long‐term AB Current smokers: 4% AAV patients vs 29% HCs	Number of teeth, cause of tooth loss, PPD, DMFT index

*Note:* The gray‐shaded line corresponds to data from the oral virome study.

Abbreviations: AAV, anti‐neutrophil cytoplasmic antibody‐associated vasculitis; AB, antibiotics; ACPA, anti‐citrullinated protein antibodies; AID, autoimmune disease; AM, all matched; BMI, body mass index; BoP, bleeding on probing; CAL, clinical attachment loss; CCP, cyclic citrullinated peptide antibodies; CODS, clinical oral dryness score; CPITN, community periodontal index of treatment needs; CRA, chronic‐established RA; CSA, clinically suspect arthralgia; DAS28, disease activity score 28; DMARD, disease‐modifying antirheumatic drug; DMFT, decayed/missing/filled teeth; G, gingivitis; GCF, gingival crevicular fluid; GI, gingival index; HC, healthy control; IBD, inflammatory bowel disease; NORA, new‐onset rheumatoid arthritis; ns, not significant; OA, osteoarthritis; PD, periodontal disease; PH, periodontally healthy; PI, plaque index; PISA, periodontal inflamed surface area; PPD, pocket probing depth; PPI, proton pump inhibitor; pSS, primary Sjögren syndrome; RA, rheumatoid arthritis; RF, rheumatoid factor; SLE, systemic lupus erythematosus; SS, Sjögren syndrome; SWS, stimulated whole saliva; SXI, shortened xerostomia inventory; UWS, unstimulated whole saliva; vs, versus.

**TABLE 2:** Sequencing approaches and diversity metrics.

AID	References	Sequencing method	α‐diversity	β − diversity
RA	Arleevskaya et al. (2022)	16S rRNA (V1‐V9 full length, Illumina)	Early‐RA vs pre‐RA with CSA vs HCs (CSA = 0): no difference	
	Corrêa et al. (2019)	16S rRNA (V4 region, Illumina)	RA vs HCs with PD: ↑ (OTUs numbers and Shannon index) RA vs HCs without PD: ↑ (OTUs numbers, Shannon and Chao‐1 indices)	RA vs HCs with PD: no difference RA vs HCs without PD: altered β‐diversity RA patients with PD cluster separately from RA patients without PD
	Cheah et al. (2022)	16S rRNA (V3‐V4 region, Illumina)	RA vs non‐RA: no difference (Chao‐1, Shannon, Simpson)	RA vs non‐RA: no difference Clustering based on PD condition only (PD vs non‐PD)
	Chen et al. (2018)	16S rRNA (V1‐V2 region, Illumina)	RA and OA vs HCs: ↑ (observed OTUs, PD whole tree) RA vs OA: no difference	RA and OA vs HCs: altered β‐diversity RA vs OA: minor but statistically significant difference
	Chen et al. (2022)	16S rRNA (V3‐V4 region, Illumina)	PD group: ↓community richness (observed bacterial features and Chao‐1) but no difference in community evenness (Shannon) in RA vs HCs AM and PH groups: no difference	AM and PD groups: difference in microbial clustering in RA vs HCs PH group: no difference
	Cheng et al. (2021)	Shotgun metagenomic sequencing, Illumina	At‐risk vs early RA and HCs: ↓ (ACE) in PH sites but no difference in diseased sites	—
	de Jesus et al. (2021)	16S rRNA (V4 region, Illumina)	RA vs HCs: no difference	RA vs HCs: significant difference
	Eriksson et al. (2022)	16S rRNA (V3‐V4 region, Illumina)	—	RA vs non‐RA: significant difference clustering based on RA condition
	Kozhakhmetov et al. (2023)	16S rRNA (Illumina, no region specified)	RA vs HCs: ↑ (Shannon, Simpson)	RA vs HCs: no significant difference
	Kroese et al. (2021)	16S rRNA (V4 region, Illumina)	Plaque, saliva and tongue coating: no difference (Shannon, zOTUs) between early RA, at‐risk, and HCs groups	Plaque: no difference between the 3 groups Saliva and tongue coating: significant difference between early RA/at‐risk RA and HCs; no difference between early RA and at‐risk RA
	Lehenaff et al. (2021)	16S rRNA (V1‐V3 region, Illumina)	RA vs non‐RA: no difference (Shannon, observed OTUs, Faith's phylogenetic diversity) Deep subgingival sites vs PH sites: ↑ in RA and non‐RA Not associated with RA status	RA vs non‐RA: no difference Deep subgingival sites vs PH sites: significant difference Clustering based on PPD and not on RA status
	Liu et al. (2020)	16S rRNA (V4 region, Illumina)	RA vs HCs: no difference in community richness (ACE, Chao‐1, Simpson) but ↑community evenness (Shannon) PD vs RA and PD vs HCs: no difference	No difference between the 3 groups
	Liu et al. (2024)	16S rRNA (V3‐V4 region, Illumina)	Active RA vs inactive RA vs HCs: no difference across the groups	Significant difference between the groups according to PCoA plots (PCoA2: HCs<active RA<inactive RA)
	Lopez‐Oliva et al. (2018)	16S rRNA (V1‐V3 and V7‐V9 regions, Illumina)	RA vs HCs: no difference (ACE)	Altered β‐diversity in RA vs HCs (in terms of community membership and structure) Clustering based on RA status
	Mikuls et al. (2018)	16S rRNA (V1‐V3 region, Illumina)	RA vs OA: no difference (observed OTUs, Chao‐1, Shannon, evenness, Good's) RA/OA without PD vs RA/OA with PD: ↑ Not impacted by RA/OA status but by PD	RA vs OA: no difference Clustering based on PD and smoking status (also ethnic origin and marital status) but not on RA‐OA status
	Scher et al. (2012)	16S rRNA (V1‐V2, pyrosequencing)	NORA vs CRA vs HCs: no difference between the 3 groups (Inverse Simpson, Shannon, Chao‐1)	NORA vs CRA vs HCs: no difference Clustering based on PD (moderate and severe forms) but not on RA status
	Tong et al. (2019)	16S rRNA (V3‐V4 region, Illumina)	At‐risk vs HCs: ↓ (ACE, Faith's phylodiversity index) At‐risk vs RA: ↓ (Shannon)	Gradual change from HCs, high‐risk to RA patients
	Zhang et al. (2015)	Shotgun metagenomic sequencing and MGWAS, Illumina	Not reported for oral samples	RA vs HCs: alteration of dental plaque and salivary community composition Clustering based on RA status
	Guo et al. (2022)	Shotgun metagenomic sequencing, Illumina	Dental plaque virome: ↓community evenness (Shannon) in untreated and treated RA patients vs. HCs, ↓community richness (observed vOTUs) in treated RA vs HC and treated vs untreated RA (no difference between untreated RA and HCs) Saliva virome: no difference in community evenness (Shannon) across groups but ↓community richness (observed vOTUs) in treated RA vs untreated RA and HCs	Dental plaque and saliva viromes: clear community differences between the 3 groups (untreated RA, treated RA, HCs)
SS	Alam et al. (2020)	16S rRNA (V1‐V3 full; pyrosequencing)	Mixed results with ↑Shannon but no difference for Chao‐1 (species richness)	Distinct β‐diversity in pSS and controls both in sicca and non‐sicca conditions
	de Paiva et al. (2016)	16S rRNA (V4, Illumina)	↓ (Shannon)	Significant difference in β‐diversity (composition of the microbiome) between SS and HCs (distinct clustering by unweighted UniFrac analysis) But lack of clustering by weighted Unifrac analysis (difference between SS and HCs primarily driven by low abundant taxa)
	Kim et al. (2022)	16S rRNA (V3‐V4, Illumina)	No difference (Shannon and Simpson) between pSS sicca and non‐SS sicca ↓ in anti‐SSA positive vs anti‐SSA negative SS groups (Shannon, Simpson)	Distinct β‐diversity between pSS sicca and non‐SS sicca Disease status accounted for 5.9% of the variation
	Li et al. (2016)	16S rRNA (V1‐V3, Illumina)	↓ (fewer genera and OTUs) but ns	—
	Martínez‐Nava et al. (2023)	16S rRNA (V3‐V4, Illumina)	↑ (Shannon)	No difference
	Rusthen et al. (2019)	16S rRNA (V3‐V5, pyrosequencing)	No difference (Chao‐1, Shannon and Simpson) between pSS, non‐SS sicca and HCs	—
	Saúco et al. (2023)	16S rRNA (V3‐V4, Illumina)	No difference (Shannon and richness indices)	No difference by SS status Distinct β‐diversity (microbial community) associated with ↓ salivary flow
	Sembler‐Møller et al. (2019)	16S rRNA (V1‐V3, Illumina)	No difference (Shannon) between pSS and non‐SS sicca	No difference between pSS and non‐SS sicca
	Sharma et al. (2020)	16S rRNA (V3‐V4, Illumina)	No difference (Chao‐1, Shannon, observed species)	—
	Siddiqui et al. (2016)	16S rRNA (V1‐V2, pyrosequencing)	↓ (Chao‐1, Shannon, observed species)	Distinct β‐diversity in pSS patients and HCs
	Tseng et al. (2021)	16S rRNA (V3‐V4, Illumina)	No difference (Chao‐1, Shannon, Simson, richness, Prelou, Goods coverage, ACE)	No difference (no discrimination between pSS and HCs)
	van der Meulen et al. (2018a)	16S rRNA (V4, Illumina)	No difference (Shannon, observed OTUs) between pSS, non‐SS sicca and HCs Trend toward lower richness and diversity in pSS and non‐SS sicca vs HCs	Difference between pSS and HCs but no difference between non‐SS sicca and pSS (greatest inter‐individual heterogeneity in pSS) Disease group (pSS, non‐SS sicca, HCs) and salivary secretion rate accounted for a similar proportion of variance (~4%) in bacterial composition between individuals
	van der Meulen et al. (2018b)	16S rRNA (V4, Illumina)	No difference (Shannon, observed OTUs) between pSS, non‐SS sicca and HCs	Altered b‐diversity in pSS vs non‐SS sicca and HCs (↑ heterogeneity in pSS and non‐SS vs HCs)
	van der Meulen et al. (2019)	16S rRNA (V4, Illumina)	↑ in SLE vs pSS patients	Significant difference (composition) between SLE and pSS Disease status accounted for 7.8%–8.8% of the variation
	Wang et al. (2022)	16S rRNA (V3‐V4, Illumina)	↓ in pSS vs HCs (richness)	Altered β‐diversity in pSS vs HCs
	Xie et al. (2024)	16S rRNA (V3‐V4, Illumina)	Mixed results with ↓ Chao‐1, observed species, and PD whole tree indices but ↑ Shannon and Simpson indices in pSS (saliva and plaque) vs HCs (saliva) Lower abundance and higher evenness of oral microbiota in pSS vs HCs	Altered β‐diversity in pSS vs HCs
	Zhou, Cai, et al. (2018)	16S rRNA (V3‐V4, Illumina)	Mixed results with ↓ Pielou, Shannon and Simpson indices (eveness and diversity) but no differences for Ace and Chao‐1 indices (richness)	No difference between pSS and HCs
	Zhou, Ling, et al. (2018)	16S rRNA (V4‐V5, Illumina)	No difference (ACE, Chao‐1, Shannon, Simpson) between pSS and HCs	Altered β‐diversity in pSS vs HCs
SLE	Corrêa et al. (2017)	16S rRNA (V4 region)	↑ in SLE vs HC without PD ↓ in SLE vs HC with PD	Significant difference between SLE and HCs SLE with PD compositionally different and more pathogenic subgingival microbiota than HC with PD (loss of heterogeneity) SLE affects microbiota independently of PD
	Li et al. (2020)	16S rRNA (V4‐V5 regions)	↓ in SLE vs HC No difference between new‐onset and treated SLE patients and between remission and active SLE	Significant difference between SLE and HCs (difference in microbial community based on Jaccard and unweighted Unifrac but ns for weighted Unifrac and Bray–Curtis dissimilarity) No difference between new‐onset and treated SLE patients and between remission and active SLE
	Liu et al. (2021)	16S rRNA (V4‐V5 regions)	↑ in SLE vs HC (for Shannon and Simpson indices, ns for ACE and Chao1 richness indices)	Significant difference between SLE and HCs
	Guo et al. (2023)	16S rRNA (V3‐V4 regions)	↑ in SLE vs HC (Shannon and Simpson indices)	Significant difference between SLE and HCs (OTU distribution)
	van der Meulen et al. (2019)	16S rRNA (V4 region)	↑ in SLE vs pSS patients	Significant difference (composition) between SLE and pSS Disease status accounted for 7.8%–8.8% of the variation
AAV	Esberg et al. (2022)	16S rRNA (V3‐V4 region)	↓ in acute AAV vs HCs	Greater β‐diversity in acute AAV vs HC (more heterogeneous and compositionally distinct salivary microbiota)

*Note:* The gray‐shaded line corresponds to data from the oral virome study.

Abbreviations: 16S rRNA, 16S ribosomal RNA; AAV, anti‐neutrophil cytoplasmic autoantibody‐associated vasculitis; AID, autoimmune disease; CRA, chronic‐established RA; HC, healthy control; MGWAS, metagenome‐wide association study; NORA, new‐onset rheumatoid arthritis; ns, not significant; OA, osteoarthritis; OTU, operational taxonomic unit; PCoA: principal coordinates analysis; PD, periodontal disease; PH, periodontally healthy; pSS, primary Sjögren's syndrome; RA, rheumatoid arthritis; SLE, systemic lupus erythematosus; SS, Sjögren syndrome; vs, versus; zOTU, zero‐radius operational taxonomic unit.

Periodontal status was clinically assessed in 12 studies (Cheah et al. 2022; Chen et al. 2022; Cheng et al. 2021; Corrêa et al. 2019; Eriksson et al. 2022; Kozhakhmetov et al. 2023; Kroese et al. 2021; Lehenaff et al. 2021; Liu et al. 2020; Lopez‐Oliva et al. 2018; Mikuls et al. 2018; Scher et al. 2012), while others relied on self‐reported oral health data (de Jesus et al. 2021; Tong et al. 2019) (Table 1). Some studies specifically examined the oral microbiota in RA patients with periodontitis (PD) (Chen et al. 2022; Corrêa et al. 2019; Lehenaff et al. 2021; Mikuls et al. 2018).

#### 3.2.2 Dental Plaque

##### 3.2.2.1 α‐Diversity

Among the 12 studies that analyzed dental plaque samples from RA patients (Table 1), α‐diversity findings were inconsistent and often influenced by periodontal status (Table 2). The majority of studies, including Cheah et al. (2022), Chen et al. (2022; in periodontally healthy individuals), Kroese et al. (2021), Mikuls et al. (2018), Lopez‐Oliva et al. (2018), Lehenaff et al. (2021), and Scher et al. (2012), found no significant differences in α‐diversity between RA and controls, including healthy individuals and OA patients (Mikuls et al. 2018). Liu et al. (2020) observed similar community richness between groups, but higher evenness in RA. Cheng et al. (2021) reported no difference at diseased sites, but a significant reduction in richness at periodontally healthy sites in early RA, suggesting that early RA‐associated shifts may be masked by local inflammation. In contrast, Chen et al. (2022) found decreased species richness in RA patients with PD, while evenness remained unchanged, reinforcing the dominant role of periodontal inflammation. Only two studies—Corrêa et al. (2019) and Kozhakhmetov et al. (2023)—reported increased α‐diversity in RA patients compared to controls. Corrêa et al. observed this trend both in individuals with and without PD, suggesting a possible influence of systemic immune dysregulation. However, Kozhakhmetov's results are more difficult to interpret due to the use of mixed oral samples (dental plaque, tongue, gum, and tonsil scrapings). Collectively, these findings suggest that α‐diversity may be more strongly shaped by local periodontal status than by RA itself, particularly in established disease.

##### 3.2.2.2 β‐Diversity (Table 2)

In some studies, β‐diversity separated RA from controls, even without PD (Corrêa et al. 2019; Lopez‐Oliva et al. 2018; Zhang et al. 2015), while other studies, including Cheah et al. (2022), Mikuls et al. (2018), Scher et al. (2012), and Lehenaff et al. (2021), found that PD, but not RA status, was the dominant factor explaining microbial variation. Chen et al. (2022) further showed that β‐diversity differences emerged only within PD subgroups, implying that RA may enhance PD‐related dysbiosis rather than drive distinct shifts on its own.

##### 3.2.2.3 Enriched and Decreased Taxa (Table 3)

**TABLE 3:** Genus‐ and species‐level alterations in the oral microbiome of patients with systemic AID.

AID	References	Increased genus	Decreased genus	Increased species	Decreased species	Comments
RA	Arleevskaya et al. (2022)	—	In early‐RA vs pre‐RA and HCs: *Porphyromonas* sp.	No difference at species level within *Porphyromonas* sp.		Also 42 established RA patients but no exhaustive analysis of oral microbiota
	Corrêa et al. (2019)	—	—	RA vs HCs without PD: *Prevotella* spp. (*P. melaninogenica, P. pallen, P. denticola, P. histicola, P* *. nigrescens* , *P. oulorum, P* *. maculosa* , *P. salivae*), *Selenomonas noxia, Selenomonas sputigena, Anaeroglobus geminatus, Corynebacteriummatruchotii* RA vs HCs with PD: *Prevotella* spp. (*P. dentalis, P. histicola, salivae*), *Aggregatibacter DQ003635, Mogibacterium timidum, Parvimonas micra, Paseudomonas aeruginosa*	RA vs HCs without PD: *Ralstonia* oral taxon 027, *Delftia tsuruhatensis, Prevotella pleuritidis, Pelmononas puraquae, Veillonella* HB016, *Sphingomonas* nbw625e08c1, *Haemophilus* 3A01, *Streptococcus anginosus, Rothia aeria, Kingella oralis, Treponema* oral taxon G53, *Streptococcus* FX003, *Haemophilus, Actinomyces meyeri…* RA vs HCs with PD: *Treponema* oral taxon G53, *Streptococcus anginosus, Prevotella marshii, Actinomyces meyerii*	↑ concentration of gram‐negative anaerobic species in RA vs HC with PD
	Cheah et al. (2022)			RA vs HCs: *Actinomyces* spp., *Cryptobacterium* spp., *Dialister* spp., *Desulfovibrio* spp., *Fretibacterium* spp., *Leptotrichia* spp., *Prevotella* spp., *Selenomonas* spp., *Treponema* spp., *Veillonellaceae [G1]* spp.	RA vs HCs: *Aggregatibacter* spp., *Gemella* spp., *Granulicatella* spp., *Haemophilus* spp., *Neisseria* spp., *Streptococcus* spp.	RA oral microbiome enriched with inflammophilic and citrulline producing species ➔ role in the production of autoantigenic citrullinated peptides
	Chen et al. (2018)	RA vs OA: *Neisseria, Haemophilus, Prevotella, Veillonella, Fusobacterium, Aggregatibacter, Actinobacillus* Arthritis (RA + OA) vs HCs: *Prevotella, Neisseria, Porphyromonas, Veillonella, Haemophilus, Rothia, Streptococcus, Actinomyces, Granulicatella, Leptotrichia, Lautropia, Fusobacterium*	RA vs OA: *Streptococcus, Actinomyces, Lautropia, Rothia, Granulicatella, Ruminococcus, Oribacterium, Abiotrophia*	RA vs OA: *Neisseria subfava, Haemophilus parainfuenzae, Veillonella dispar, Prevotella tannerae, Actinobacillus parahaemolyticus* Arthritis (RA + OA) vs HCs: *Prevotella melaninogenica, Veillonella dispar*	RA vs OA: *Rothia dentocariosa, Ruminococcus gnavus*	
	Chen et al. (2022)	—	—	AM group: *Streptococcus anginosus, Treponema denticola, unidentified* *Fusobacterium species* *Aminipila butyrica* and *Peptococcus simiae*: positive correlation with ACPA levels PD group: *S. anginosus* *and 3 unidentified species of genera Actinomyces, Fusobacterium and Parvimonas* *Aminipila butyrica* and *Peptococcus simiae*: positive correlation with ACPA levels	AM group: *Haemophilus parainfluenzae, Streptococcus sanguinis* PD group: *Pseudomonas batumici*	
	Cheng et al. (2021)	At‐risk vs HCs: *Bifidobacterium* (PH sites), *Porphyromonas* (PH and PD sites) Early‐RA: *Cardiobacterium* (PH and PD sites), *Capnocytophaga* (diseased sites), *Neisseria* (PD sites), *Streptococcus* (PD sites)	At‐risk and early‐RA vs HC: *Veillonella* (PH sites)	At‐risk vs HCs: *Arthrobacter chlorophenolicus, Porphyromonas gingivalis* (PH and PD sites)		
	de Jesus et al. (2021)	RA vs non‐RA (at‐risk): *Streptococcus, Rothia, Leptotrichia*	RA vs non‐RA: *Fusobacterium, Porphyromonas, Aggregatibacter, Capnocytophaga*	RA vs non‐RA: *Streptococcus salivarius, Rothia mucilaginosa, Prevotella* sp. HMT_300, *Leptotrichia* sp.HMT_498 and HMT_221, *Selenomonas fueggei*	RA vs non‐RA: *Prevotella melaninogenica, Bacteroidetes_G5 HMT_505, Leptotrichia* sp. *HMT_392 and HMT_215, Treponema* sp. *HMT_246, Fusobacterium periodonticum, Granulicatella elegans, Veillonella parvulla, Porphyromonas endodontalis, Prevotella* sp. *HMT_304*	Also study of bitter taste receptor T2R38 polymorphisms and their association with RA
	Eriksson et al. (2022)	—	—	*Granulicatella* sp. (ASV687), *Veillonella* sp. (ASV339), *Fusobacterium nucleatum* (ASV192), *Megasphaera* sp. (ASV748), *Aquabacterium* sp. (ASV435 and ASV649), *Sphingobium* sp. (ASV435), *Novosphingobium* sp. (ASV474), *Sphingomonas insulae* (ASV626), *Novosphingobium aromaticivorans* (ASV668), *Acidovorax delafieldii* (ASV743), *Sphingomonas echinoides* (ASV993), *Sphingomonas melonis* (ASV1062)	*Alloprevotella* sp. (ASV503), *Prevotella* sp. (ASV458), *Haemophilus* sp. (ASV557), *Actinomyces* sp. (ASV991), *Streptobacillus* sp. (ASV84), *Vampirovibrio* sp. (ASV1262)	
	Kozhakhmetov et al. (2023)	RA vs HCs: *Prevotella, Leptotrichia, Neisseria, Rothia, Granulicatella, Olsenella*,	RA vs HCs: *Butyricicoccus, Cutibacterium, Ruminococcus, Subdoligranulum, Faecalibacterium, Blautia, Roseburia*	RA vs HCs: *Prevotella histicola, Porphyromonas pasteri, Leptotrichia* sp., *Actinomyces* sp., *Haemophilus* sp., *Megasphaera micronuciformis, Neisseria elongata, Olsenella genomosp. C1*	RA vs HCs: *Eubacterium coprostanoligenes, Eubacterium hallii*	↓ content of butyrate and propionate‐producing bacteria in RA vs HCs RA patients in remission: higher abundance of *Treponema* sp. And *Absconditabacteriales* (SR1) RA patients with low disease activity: higher levels of *Porphyromonas* RA patients with high RA activity: higher levels of *Staphylococcus*
	Kroese et al. (2021)	—	—	Early RA and at‐risk RA vs HCs: *Prevotella salivae* (zOTU 25; saliva), *Prevotella* sp. (zOTU 10; saliva), *Veillonella* sp. (zOTU 4; saliva + tongue coating)	Early RA and at‐risk RA vs HCs: *Neisseria flavescens/subflava* (zOTU 7); saliva + tongue coating, P*orphyromonas pasteri/* sp. oral taxon 278 (zOTU 15; saliva), *Veillonella parvula* (zOTU 12; saliva), *Streptococcus dentisani/infantis/mitis/oralis/*sp. oral taxon 058 (zOTU 1; tongue coating) Early RA vs at‐risk RA and HCs: *Fusobacterium periodonticum* (zOTU 13, saliva) Early RA vs HCs: *Fusobacterium periodonticum* (zOTU 13; tongue coating)	
	Lehenaff et al. (2021)			RA vs non‐RA: *Actinomyces meyeri, Streptococcus parasanguinis* (PH and deep subgingival sites)	RA vs non‐RA: *Gemella morbillorum, Kingella denitrificans, Prevotella melaninogenica, Leptotrichia* sp. (PH and deep subgingival sites)	Also comparison of subgingival microbiome between PH sites and deep subgingival sites
	Liu et al. (2020)	RA vs HCs: *Treponema*				Also studied the profile of periodontal pathogens (RA vs non‐RA: ↑*Tanarella, Porphyromonas, Prevotella*; ↓*Streptococcus*)
	Liu et al. (2024)	RA vs HCs: *Prevotella, Veillonella*	RA vs HCs: *Streptococcus, Fusobacterium*	RA vs HCs: *Prevotella histicola* Active RA vs inactive RA: *Neisseria perflava* Inactive RA vs active RA: *Prevotella histicola, Prevotella melaninogenica*	RA vs HCs: *Fusobacterium periodonticum*	
	Lopez‐Oliva et al. (2018)	—	—	RA vs HCs: *Actinomyces* spp., *Cryptobacterium* spp., *Dialister* spp., *Desulfovibrio* spp., *Fretibacterium* spp., *Leptotrichia* spp., *Prevotella* spp., *Selenomonas* spp., *Treponema* spp., *Veillonellaceae [G1], Cryptobacterium curtum*	RA vs HCs: *Aggregatibacter* spp., *Gemella* spp., *Granulicatella* spp., *Hemophilus* spp., *Neisseria* spp., *Streptoccocus* spp.	
	Mikuls et al. (2018)	—	RA with PD vs OA with PD: *Prevotella* RA without PD vs OA without PD: —	RA with PD vs OA with PD: *Catonella* sp. (OTU 451), *Clostridiales* sp. (OTU 85), *Lachnospiraceae* sp. (OTU 96), *Peptostreptococcaceae* sp. (OTU 495), *Porphyromonas* sp. (OTU 285), *Prevotella multiformis* (OTU 685), *Prevotella* sp. (OTU 443), *Treponema* sp. (OTUs 230 and 236) RA without PD vs OA without PD: /	RA with PD vs OA with PD: — RA without PD vs OA without PD: *Streptococcus* sp. (OTU 486)	
	Scher et al. (2012)	RA vs HCs: *Anaeroglobus* NORA vs HCs: *Anaeroglobus, Phocaeiola*	RA vs HCs: *Corynebacterium, Streptococcus* NORA vs HCs: *Corynebacterium, Mitsuokella, Streptococcus* NORA vs CRA: *Mitsuokella*	RA vs HCs: *Anaeroglobus* spp. (OTU 99), *Prevotella* spp. (OTU 31, 60, 134), *Selenomonas* spp. (OTU168) NORA vs HCs: *Prevotella* spp. (OTU 60), *Leptotrichia* spp. (OTU 87) (irrespective of PD status, completely absent from HCs microbiota), *Anaeroglobus* spp. (OTU 99), *Selenomonas* spp. (OTU 168), *Prevotella* spp. (OTU 31, 134), *Phocaeiola* spp. (OTU92), *Neisseria* spp. (OTU16), *Porphyromonas* spp. (OTU1) NORA vs CRA: *Tanarella forsythia* spp. (OTU 13), *Treponema medium* (OTU 32), *Porphyromonas* spp. (OTU 57), *Selenomonas* spp. (OTU 231), *Prevotella* spp. (OTU 26)	RA vs HCs: *Leptotrichia* spp. (OTU 9, 12, 86), *Corynebacterium* spp. (OTU 4), *Capnocytophaga* spp. (OTU 74) NORA vs HCs: *Leptotrichia* spp. (OTU 12), *Corynebacterium* spp. (OTU 4), Unclassified TM7 (OTU 58) NORA vs CRA: Prevotella spp. (OTU 39)	
	Tong et al. (2019)	RA vs at‐risk and HCs: *Actinomyces, Treponema_2, Selenomonas, Prevotella_6, Megasphaera, Atopobium* At‐risk vs RA: *Rothia* At‐risk vs RA and HCs: *Gemella*	At‐risk and RA vs HC: *Neisseria, Porphyromonas, Haemophilus, Filifactor*	At‐risk vs RA: *uncultured bacteria Rothia, uncultured bacteria Porphyromonas*	At‐risk and RA vs HCs: *Defluviitaleaceae* UCG‐011 At‐risk vs HCs: *Porphyromonas gingivalis*	
	Zhang et al. (2015)	RA vs HCs: *Veillonella* (dental plaque, saliva), *Atopobium*	RA vs HCs: *Haemophilus, Aggregatibacter, Cardiobacterium, Eikenella, Kingella* (dental plaque, saliva)	RA vs HCs: *Rothia mucilaginosa, Lactobacillus salivarius, Atopobium* spp., *Cryptobacterium curtum* (dental plaque, saliva), *Veillonella* sp., *Selenomonas flueggei* (saliva), *Actinomyces* sp. (dental plaque), *Rothia dentocariosa* ,	RA vs HCs: *Haemophilus* spp. *Porphyromonas gingivalis, Kingella denitrificans* (dental plaque, saliva), *Rothia aeria, Lactococcus* sp., *Cardiobacterium hominis* (saliva), *Aggregatibacter* sp., *Neisseria* spp., *Prevotella intermedia* (dental plaque), *Neisseria* spp., *Capnocytophaga ochracea, Leptotrichia sp*	RA status has the strongest effect on dental and salivary microbiomes among all available phenotypes DMARD treatment partially restored the oral microbiome toward HC‐like composition Also fecal samples
	Guo et al. (2022)			Treated RA vs untreated RA and HCs: *Lactococcus phage* vOTU70 (dental plaque)	Untreated RA vs treated and HCs: *Lactococcus phage* vOTU70 (saliva)	
SS	Alam et al. (2020)	pSS vs controls: (5 genera) *Streptococcus, Prevotella, Lactobacillus, Atopobium, Staphylococcus*	pSS vs controls: (34 genera) *Haemophilus, Neisseria, Lautropia, Leptotrichia, Fusobacterium*	pSS vs controls: *Streptococcus*_HQ748137_s, *Prevotela histicola, Streptococcus*_HQ762034_s, *Streptococcus parasanguinis, Streptococcus*_4P003152_s*…* pSS without dryness vs HCs *Rothia mucilaginosa, Granulicatella adiacens, Prevotella melaninogenica, Prevotella histicola, Streptococcus*_HQ762034_s… pSS with dryness vs non‐SS sicca: *Veillonella dispar, Prevotella histicola, Streptococcus*_4P003152_s, *Veillonella parvula, Lactobacillus salivarius…*	pSS vs controls: *Haemophilus parainfluenzae, Neisseria perflava, Neisseria subflava, Fusobacterium periodonticum, Lautropia mirabilis…* pSS without dryness vs HCs: *Haemophilus parainfluenzae, Campylobacter concisus, Veillonella rogosae, Haemophilus sputorum, Lautropia mirabilis…* pSS with dryness vs non‐SS sicca: *Haemophilus parainfluenzae, Neisseria perflava, Neisseria subflava, Streptococcus*_CP006776_s, *Fusobacterium periodonticum…*	Logistic regression analysis: ‐ Strong association of *Prevotella melaninogenica* with pSS (OR = 22.4)—Reduced presence of *Veillonella rogosae and Eikenella corrodens* in pSS (OD = 0.006)—Very strong association between *Haemophilus_*HQ807753_s and pSS in the non‐sicca comparisons (OD = 1.3 × 10^16^) ‐↓ presence of *Neisseria_uc* in pSS in the *sicca comparison* (OD = 0.006)
	de Paiva et al. (2016)	SS vs HCs: *Streptococcus*	SS vs HCs: *Leptotrichia, Fusobacterium* and the low‐abundance genera *Bergeyella, Peptococcus, Butyrivibrio*	—	—	Also analysis of the stool and conjunctiva microbiomes
	Kim et al. (2022)	pSS vs non‐SS sicca: *Streptococcus* « Dysbiosis cluster » observed in some pSS subjects: *Streptococcus*	pSS vs non‐SS sicca: *Lactobacillus*	« Dysbiosis cluster » observed in some pSS subjects: *Streptococcus oralis*	pSS vs non‐SS sicca: *Bacteroides vulgatus, Staphylococcus aureus, Methylorubrum extorquens, Blautia coccoides, Lactobacillus johnsonnii* « Dysbiosis cluster » observed in some pSS subjects: *Bacteroides vulgatus, Bifidobacterium longum, Faecalibacterium prausnitzii*	PCA analysis: disease status contributed to 5.9% of saliva (parotid gland) microbiota composition variation “Dysbiosis cluster”
	Li et al. (2016)	pSS vs HCs: *Leucobacter, Delftia, Pseudochrobactrum, Ralstonia, Mitsuaria*	pSS vs HCs: *Haemophilus, Neisseria, Comamona, Granulicatella, Limnohabitans*	—	—	The impact of prednisone on oral microbiota composition was also assessed
	Martínez‐Nava et al. (2023)	pSS vs HCs: *Prevotella, Streptococcus, Veillonella, Fusobacterium, Leptotrichia, Oribacterium, Actinomyces, Porphyromonas* After age and salivary flow adjustment: 16 ASVs significantly associated with pSS (*Prevotella*: 6 ASVs, *Gamella*: 2 ASVs, *Veillonella, Actinobacillus, Corynebacterium, Rothia, Fretibacterium, Oribacterium*: 1 ASV)	pSS vs HCs: *Selenomonas*	—	—	The oral microbiota composition was also analyzed in relation to anti‐SSA/SSB antibody status, and the presence of parotid gland enlargement
	Rusthen et al. (2019)	—	pSS and non‐SS sicca vs HCs: *Haemophilus, Neisseria*	—	pSS vs HCs: *Porphyromonas pasteri* pSS and non‐SS sicca with normal salivation vs HCs: *Actinomyces lingnae, Fusobacterium nucleatum subsp. Vincentii, Lachnoanaerobaculum orale, Megasphaera micronuciformis, Oribacterium asaccharolyticum, Prevotella nanceiensis, Stomatobaculum longum, Streptococcus intermedius* pSS vs non‐SS sicca: *Granulicatella adiacen* pSS vs non‐SS sicca with hyposalivation: *Capnocytophaga leadbetteri, Granulicatella* *adiacens, Neisseria* *flavescens, Prevotella* *nanceiensis, RuminococcaceaeG1spt*	
	Saúco et al. (2023)	*Prevotella*	*Streptococcus*	—	—	Paper analyzing the relationship between oral microbiota and hyposalivation in aging women
	Sembler‐Møller et al. (2019)	No significant difference between pSS and non‐SS sicca				
	Sharma et al. (2020)	*Bifidobacterium, Lactobacillus, Dialister*	*Leptotrichia*	—	—	↑a‐diversity in pSS with renal tubular acidosis (RTA) vs pSS without RTA ↓ a‐diversity in pSS treated with steroids vs no steroids
	Siddiqui et al. (2016)	*Streptococcus, Veillonella*	*Treponema, Peptostreptococcaceae*_[XIII][G‐1], *Bacteroidaceae*_[G‐1], *Moryella, Fretibacterium, Porphyromonas, Tannerella, Catonella*	*Veillonella* sp._Oral_Taxon_917	*Treponema* sp._Oral_Taxon_237, *Peptostreptococcaceae*_[XIII][G‐1] sp._Oral_Taxon_113, *Bacteroidaceae*_[G‐1] sp._Oral_Taxon_272, *Moryella* sp._Oral_Taxon_373, *Fretibacterium* multispecies_spp23_2, *Prevotella nanceiensis, Tannerella forsythia, Catonella morbi, Fusobacterium periodonticum, Prevotella pallens*	
	Tseng et al. (2021)	*Megasphaera*	*Haemophilus, Aggregatibacter, Abiotrophia, Cardiobacterium, Johnsonella, Bifidobacterium*	*Actinomyces odontolyticus, Atopobium parvulum, Corynebacterium argentoratense, Megasphaera micronuciformis, Peudomonas geniculate, Rothia amarae*	*Haemophilus parainfluenzae, Aggregatibacter aphrophilus* *Abiotrophia defectiva* *Neisseria elongata, Capnocyophaga granulosa, Granulicatella elegans, Leptotrichia shahii, Cardiobacterium hominis, Leptotrichia goodfellowii, Campylobacter gracilis, Johnsonella ignava, Kingella denitrificans, Haemophilus pittmaniae, Kingella potus, Neisseria baciliformis, Leptotrichia trevisanii, Prevotella saccharolytica*	
	van der Meulen et al. (2018a)	pSS vs HCs: *Alloscardovia, Bifidobacterium, Scardovia, Atopobium, Lactobacillus, Parvimonas, Peptostreptococcaceae, Anaeroglobus, Dialister* pSS vs HCs after adjusting for SWS: *Scardovia, Anaeroglobus, Bifidobacterium, Parvimonas*	pSS vs HCs: *Alloprevotella, Bergeyella, Abiotrophia, Granulicatella, Enterococcus, Ruminococcaceae_G1, Lautropia, Neisseria, Haemophilus* pSS vs non‐SS sicca: *Bergeyella, Granulicatella* pSS vs HCs after adjusting for SWS: *Abiotrophia, Granulicatella, Enterococcus, Bergeyella, Ruminococcaceae_G1*			
	van der Meulen et al. (2018b)	pSS vs HCs: *Fusobacterium, Selenomonas* pSS vs non‐SS patients: *Abiotrophia, Shuttleworthia*	pSS vs HCs: *Streptococcus*, *Haemophilus*	—	—	↓ *Streptococcus* relative abundance: disease specific and not related to SWS, in contrast with previous studies *Haemophilus, Neisseria, Lactobacillus* relative abundance: significantly correlated with SWS, but not disease status
	van der Meulen et al. (2019)	In SLE vs pSS: *Actinomyces, Granulicatella, Prevotella, Capnocytophaga, Corynebacterium, Stomatobaculum, Cardiobacterium*	In SLE vs pSS: *Lactobacillus*	—	—	Also fecal samples Oral status (number of teeth 15%, self‐reported condition of the gums 16%, xerostomia 10%) contributes more to oral microbiota composition than disease phenotype (7.8%–8.8%)
	Wang et al. (2022)	*Oribacterium, Capnocytophaga, Sphingomonas, Granulicatella, Veillonella, Leptotrichia, Prevotella*	*[Eubacterium]_nodatum, [Eubacterium]_saphenum, Corynebacterium, Catonella, Filifactor, Dialister, Bacteroides, Actinobacillus, Treponema, Aggegatibacter, Lautropia, Haemophilus, Neisseria*	—	—	Also anal swabs, vaginal swabs, salivary gland tissues, and peripheral blood samples Study of microbial composition after HCQ treatment
	Xie et al. (2024)	Saliva: *Prevotella, Veillonella, Alloprevotella, Rothia*	Saliva: *Leptotrichia, Fusobacterium, Corynebacterium, Actinomyces, Campylobacter*	Saliva: *Prevotella melaninogenica*	Saliva: *Fusobacterium periodonticum, Campylobacter graciis*	No plaque samples for HCs
	Zhou, Ling, et al. (2018)	*Veillonella*	*Actinomyces, Haemophilus, Neisseria, Rothia, Porphyromonas, Peptostreptococcus*	—	—	*Streptococcus* and *Lactobacillus* (cariogenic bacteria): no significant difference between pSS and HCs
	Zhou, Cai, et al. (2018)	*Prevotella, Bacteroides, Coriobacteria, Atopobium, Megasphaera, Peptostreptococcus, Shuttleworthia*	*Neisseria, Streptococcus, Eikenella, Comamonas, Johnsonella, Lautropia, Aquabacterium*	—	—	
SLE	Corrêa et al. (2017)	/	Non‐PD group: *Sphingomonas* PD group: *Clostridiales*	Non PD group: *Prevotella nigrescens, oulorum and oris, Lachnospiraceae A IR009, Selenomonas noxia* PD group: *Prevotella oulorum and pleuritidis, Pseudomonas* spp., *Treponema maltophilum, Actinomyces IP073, Fretibacterium fastidiosum, Fusobacterium oral taxon 360,450, Anaeroglobus geminatus, TM7* oral taxon 437	PD group: *Rothia aeria, Capnocytophaga gingivalis, Rasltonia oral taxon 027, Leptotrichia oral taxon A71, Streptococcus sanguinis, Haemophilus parainfluenzae*	
	Li et al. (2020)	In SLE vs HC: *Barnesiella*, *Blautia, Lactobacillus, Pyramidobacter, Veillonella*	In SLE vs HC: *Mobiluncus, Corynebacterium, Rothia, Tannerella, Chryseobacterium* *Oribacterium* *Parvimonas, Filifactor, Aquabacterium, Caulobacter, Acinetobacter, Eikenella, Halomonas, Methylobacterium, Nevskia, Novosphingobium, Kingella, Pelagibacterium, Phyllobacterium, Stenotrophomonas, Variovorax, Zoogloea, Mycoplasma* In active vs remission SLE: *Haemophilus*	In SLE vs HC: *Lactobacillus mucosae, Prevotella enoeca, Ruminococcus* sp. *5 1 39BFAA, uncultured Oribacterium* sp., *Comamonas testosteroni, Pyramidobacter piscolens*	In SLE vs HC: *Corynebacterium durum, Corynebacterium matruchotii, uncultured Capnocytophaga* sp., *Chryseobacterium taiwanense, Lachnospiraceae_bacterium feline oral taxon 001, Johnsonella ignava, Eubacterium* sp. *oral clone DO008, Eubacterium brachy, uncultured Oribacterium* sp., *Filifactor alocis, uncultured Clostridiales bacterium, Dialister invisus, Nevskia aquatilis, Caulobacter* sp., *Pseudomonas graminis, Eikenella* sp. *NML99‐0057, Treponema denticola, Treponema lecithinolyticum, Mycoplasma ovis* In active vs remission SLE: uncultured *Bacteroidetes* *bacterium, Prevotella nanceiensis*	
	Liu et al. (2021)	In SLE vs HCs: *Selenomonas, Prevotella, Veillonella, Lautropia, Fusobacterium*	In SLE vs HCs: *Streptococcus, Rothia, Actinomyces, Gemellacea, Granulicatella*	—	In SLE vs HCs: *Streptococcus anginosus*	Also fecal samples Disease phenotype explained 10.40% of the variation in the overall bacterial composition of saliva between SLE and HC
	Guo et al. (2023)	In SLE vs HCs: 28 genera increased, including *Prevotella, Veillonella, Leptotrichia, Alloprevotella, Lachnoanaerobaculum, Campylobacter, Selenomonas* In post‐treatment stable SLE vs HC: 21 genera increased including the same as above	In SLE vs HCs: 22 genera deceased, including *Streptococcus, Porphyromonas, Rothia, Haemophilus, Granulicatella, Halomonas, Peptostreptococcus* In post‐treatment stable SLE vs HC: 21 genera decreased including the same as above + *Actinomyces*	—	—	Correlation between ↑disease activity, ↑of *Abiotrophia* and Lactobacillales_P5D1–392 and of *Phyllobacterium* and ↓*Micrococcaceae*_unclassified Development of an oral microbiota diagnostic model of SLE
	van der Meulen et al. (2019)	In SLE vs pSS: *Actinomyces, Granulicatella, Prevotella, Capnocytophaga, Corynebacterium, Stomatobaculum, Cardiobacterium*	In SLE vs pSS: *Lactobacillus*	—	—	Also fecal samples Oral status (number of teeth 15%, self‐reported condition of the gums 16%, xerostomia 10%) contributes more to oral microbiota composition than disease phenotype (7.8%–8.8%)
AAV	Esberg et al. (2022)	*Lactobacillus, Staphylococcus, Arthrospira, Ruminococcaceae G‐1, Cardiobacterium*, *Scardovia, Fretibacterium, Veillonellaceae G‐1*	*Haemophilus, Fusobacterium, Alloprevotella, Schaalia, Leptotrichia, TM7 G1, Stomatobaculum, Megasphaera, Lachnoanaerobaculum*	15 species including *Campylobacter gracilis, Arthrospira platensis, Lactobacillus fermentum, Treponema socranskii, Lactobacillus paracasei, Scardovia wiggsiae, Oribacterium* sp. HMT078, *Streptococcus mutans*	76 species including *Fusobacterium periodonticum, Solobacterium moorei, Peptostreptococcaceae* [XI][G‐1] *sulci, Leptotrichia*sp. HMT 417, *Stomatobaculum sp*. HMT 097, *Haemophilus parainfluenzae, Saccharibacteria* (TM7) [G‐1] *bacterium* HMT 352, *Mogibacterium diversum*	

*Note:* The gray‐shaded line corresponds to data from the oral virome study.

Abbreviations: AAV, anti‐neutrophil cytoplasmic autoantibody‐associated vasculitis; ACPA, anti‐citrullinated protein antibodies; AID, autoimmune disease; AM, all matched; ASV, amplicon sequence variant; CRA, chronic‐established RA; HC, healthy control; HCQ, hydroxychloroquine; NORA, new‐onset rheumatoid arthritis; OA, osteoarthritis; OTU, operational taxonomic unit; PD, periodontal disease; PH, periodontally healthy; pSS, primary Sjögren's syndrome; RA, rheumatoid arthritis; RTA, renal tubular acidosis; SLE, systemic lupus erythematosus; sp., species; SS, Sjögren syndrome; SWS, stimulated whole saliva; vs, versus.

Taxonomic findings were variable, with no consistently replicated RA‐specific microbial signature in the subgingival niche. However, many cohorts showed enrichment of anaerobic and pro‐inflammatory species, particularly from *Prevotella* (e.g., *P. melaninogenica, histicola, and denticola*), *Treponema, Selenomonas, Veillonella, Fretibacterium and Dialister* genera (Cheah et al. 2022; Corrêa et al. 2019; Kozhakhmetov et al. 2023; Liu et al. 2020; Lopez‐Oliva et al. 2018; Scher et al. 2012; Zhang et al. 2015). However, these taxa are also commonly associated with PD and their enrichment may reflect differences in oral health status rather than RA‐specific mechanisms. Conversely, some studies reported depletion of commensals (*Haemophilus, Neisseria*, *Granulicatella*) (Cheah et al. 2022; Chen et al. 2022; Corrêa et al. 2019; Kroese et al. 2021; Lopez‐Oliva et al. 2018; Mikuls et al. 2018; Scher et al. 2012; Zhang et al. 2015), though again not consistently. Importantly, studies that stratified or adjusted for PD, such as Mikuls et al. (2018) and Lehenaff et al. (2021), found no significant taxonomic differences between RA and controls, suggesting that periodontal inflammation may underlie many of the observed microbial shifts. Notably, one study reported increased detection of *Pg* in at‐risk ACPA‐positive individuals, even at periodontally healthy sites (Cheng et al. 2021). Taken together, the variability in taxonomic findings, along with differences in PD status, control group selection (e.g., OA in Mikuls et al. 2018) and medications, suggests that microbial shifts observed in RA dental plaque are not uniformly disease‐specific, and may instead reflect a complex interplay between systemic immune status, local inflammation, and oral health.

#### 3.2.3 Saliva

##### 3.2.3.1 α‐Diversity

α‐diversity findings were inconsistent across the five studies that analyzed the bacterial salivary microbiota in RA patients (unstimulated saliva: Chen et al. 2018; Tong et al. 2019; Zhang et al. 2015; stimulated saliva: Eriksson et al. 2022; Kroese et al. 2021). Tong et al. (2019) observed significantly reduced microbial richness and diversity in at‐risk ACPA‐positive individuals, compared to both RA patients and HCs, suggesting early ecological disruption in the preclinical phase (Table 2). In contrast, Chen et al. (2018) found increased α‐diversity in RA patients compared to healthy and OA controls, although periodontal status was not assessed. Finally, Kroese et al. (2021) reported no significant differences between RA patients and HCs.

##### 3.2.3.2 β−Diversity (Table 2)

In contrast, β‐diversity analyses consistently indicated that salivary microbial community composition was altered in RA. RA patients (Chen et al. 2018; Eriksson et al. 2022; Kroese et al. 2021; Tong et al. 2019; Zhang et al. 2015), as well as at‐risk individuals (Kroese et al. 2021; Tong et al. 2019) showed distinct clustering from HCs, suggesting that shifts in microbial composition may accompany or even precede the onset of clinical RA. These differences were observed regardless of whether the control groups comprised healthy individuals (Chen et al. 2018; Kroese et al. 2021; Tong et al. 2019; Zhang et al. 2015) or non‐RA disease controls such as OA patients (Chen et al. 2018). Notably, Tong et al. (2019) described a gradual shift in salivary microbiota profiles from HCs to at‐risk individuals and then to RA patients, supporting a potential progressive trajectory of dysbiosis during disease development. In Eriksson et al. (2022), where periodontal status was considered, RA status still contributed to microbial differences, although the relative influence of systemic inflammation versus local oral disease remains difficult to discriminate.

##### 3.2.3.3 Enriched and Decreased Taxa

RA was associated with a shift in salivary microbiota from commensals toward anaerobic and inflammophilic genera, although the specific taxa varied between cohorts and disease stages (Table 3). Genera commonly enriched in RA or at‐risk individuals included *Prevotella* (Chen et al. 2018; Kroese et al. 2021; Tong et al. 2019), *Veillonella* (Chen et al. 2018; Kroese et al. 2021; Zhang et al. 2015), *Actinomyces, Selenomonas, Treponema* (Tong et al. 2019), or *Atopobium* (Tong et al. 2019; Zhang et al. 2015). In contrast, genera such as *Neisseria* (Kroese et al. 2021; Tong et al. 2019; Zhang et al. 2015), *Haemophilus* (Eriksson et al. 2022; Zhang et al. 2015), and *Granulicatella* (Chen et al. 2018) species were frequently depleted in RA or at‐risk individuals. Interestingly, *Pg* was not enriched in RA saliva and was even reduced in RA patients (Zhang et al. 2015) and in at‐risk individuals (Tong et al. 2019), suggesting niche specificity, possibly more relevant in subgingival plaque than in saliva.

#### 3.2.4 Buccal Swabs

##### 3.2.4.1 α‐Diversity (Table 2)

Among the five swab‐based studies (buccal swabs: Arleevskaya et al. 2022; de Jesus et al. 2021; dorsal tongue: Kroese et al. 2021; Liu et al. 2024; tongue, gums and tonsils swabs: Kozhakhmetov et al. 2023), only Kozhakhmetov et al. (2023) reported increased α‐diversity in RA patients compared to HCs. However, their analysis included a mixture of oral sites, which may limit direct comparison with strictly buccal swab studies. In contrast, the other studies found no statistically significant differences across groups including RA patients (both active and inactive phases; Liu et al. 2024), at‐risk or pre‐RA individuals (Arleevskaya et al. 2022; Kroese et al. 2021) and HCs.

##### 3.2.4.2 β‐Diversity

Significant differences in microbial composition were observed in tongue swabs by Liu et al. (2024), who reported distinct clustering between active RA, inactive RA, and HCs, and by Kroese et al. (2021), who found separation between at‐risk individuals and controls. In buccal swabs, distinct clustering was also observed between RA patients and HCs (de Jesus et al. 2021; Table 2). In contrast, two studies found no significant β‐diversity differences, either between RA and HCs (Kozhakhmetov et al. 2023) or across early RA, pre‐RA, and HCs (Arleevskaya et al. 2022).

##### 3.2.4.3 Enriched and Decreased Taxa

Taxonomic profiles revealed some consistent trends, though variation across studies suggests context‐ and niche‐dependent effects. Notably, *Porphyromonas* was significantly depleted in RA patients (including early RA) compared to HCs, but also to pre‐RA individuals with clinically suspect arthralgia (Arleevskaya et al. 2022; de Jesus et al. 2021; Table 3). Similarly, *Fusobacterium* (including *Fusobacterium periodonticum* ) was consistently reduced in RA, even in early stages (de Jesus et al. 2021; Kroese et al. 2021; Liu et al. 2024) in both buccal and tongue swabs. In contrast, *Streptococcus* showed a more variable pattern: it was enriched in buccal swabs (de Jesus et al. 2021) but depleted in tongue swabs (Liu et al. 2024) from RA patients, highlighting possible niche effects.

#### 3.2.5 Oral Virome

Only one study profiled the oral virome using Shotgun metagenomics (Guo et al. 2022) (Table 2).

##### 3.2.5.1 Subgingival Dental Plaque

Viral community evenness was significantly reduced in both untreated and treated RA patients compared to HCs, while viral richness was lower only in treated RA compared to both untreated and HCs, suggesting that virome dysbiosis emerges early in RA, with further loss of diversity potentially associated with immunosuppressive therapy. β − diversity analysis showed clear separation among the three groups, confirming the influence of both RA and treatment (Table 2). Notably, *Lactococcus* phage vOTU70 was enriched in treated RA patients, but nearly absent in both untreated RA patients and controls, potentially reflecting treatment effects (Table 3).

##### 3.2.5.2 Saliva

In saliva, a different pattern emerged. *Lactococcus* phage vOTU70 was abundant in HCs, depleted in untreated RA patients, and restored to near‐control levels in treated RA patients, suggesting that RA‐related depletion may be partially reversed by immunosuppressive therapy (Table 3). α‐diversity findings were more modest in saliva: evenness did not differ significantly between groups, while viral richness was reduced only in treated RA patients, mirroring dental plaque findings. β‐diversity analysis again showed significant separation among HCs, untreated and treated RA patients, with the saliva virome appearing particularly sensitive to RA disease activity (Table 2).

### 3.3 Sjögren Syndrome

#### 3.3.1 Study Characteristics

Eighteen case–control studies profiled 544 pSS patients, typically women, and spanned Europe, Asia, and the Americas (Table 1). Fifteen studies compared pSS patients with HCs (*n* = 296) (Alam et al. 2020; de Paiva et al. 2016; Li et al. 2016; Martínez‐Nava et al. 2023; Rusthen et al. 2019; Saúco et al. 2023; Sharma et al. 2020; Siddiqui et al. 2016; Tseng et al. 2021; van der Meulen et al. 2018a, 2018b; Wang et al. 2022; Xie et al. 2024; Zhou, Cai, et al. 2018; Zhou, Ling, et al. 2018), and seven studies included non‐SS sicca controls (*n* = 278; Alam et al. 2020; Kim et al. 2022; Rusthen et al. 2019; Sembler‐Møller et al. 2019; van der Meulen et al. 2018a, 2018b; Wang et al. 2022), to separate effects of hyposalivation from autoimmunity (Table 1). One study compared pSS with SLE without a HC arm (van der Meulen et al. 2019). All studies used 16S rRNA gene sequencing (Table 2). Saliva was analyzed in nine studies, oral washings in five studies, swabs in four studies and GCF in one study (Table 1). None of the included studies directly compared periodontal microbiota (dental plaque) between pSS patients and HCs. In Xie et al. (2024), dental plaque samples were collected exclusively from pSS patients (no corresponding samples from HCs).

#### 3.3.2 Saliva and Oral Washings

##### 3.3.2.1 α‐Diversity

α‐diversity findings were heterogeneous across the 14 studies that characterized the microbiome in saliva (Kim et al. 2022; Rusthen et al. 2019; Saúco et al. 2023; Sembler‐Møller et al. 2019; Sharma et al. 2020; Siddiqui et al. 2016; Tseng et al. 2021; Xie et al. 2024; S. Zhou, Cai, et al. 2018) and oral washings (Alam et al. 2020; van der Meulen et al. 2018a, 2018b; Wang et al. 2022; Zhou, Ling, et al. 2018). Some reports found reduced α‐diversity in pSS compared with HCs (Siddiqui et al. 2016; Wang et al. 2022; Table 2). However, most studies found no significant differences or index‐dependent shifts. Similarly, when pSS patients were compared with non‐SS sicca controls, most studies reported no significant differences (Kim et al. 2022; Rusthen et al. 2019; Sembler‐Møller et al. 2019; van der Meulen et al. 2018b), although Kim et al. (2022) found reduced α‐diversity in SS patients with anti‐SSA/Ro autoantibodies or with sialectasis, relative to their respective negative subgroups.

##### 3.3.2.2 β‐Diversity

The results for β‐diversity were mixed. Several studies (Alam et al. 2020, 202; Kim et al. 2022; Siddiqui et al. 2016; van der Meulen et al. 2018b; Wang et al. 2022; Xie et al. 2024; Zhou, Cai, et al. 2018) reported significant compositional differences between pSS and comparators, whereas other studies, notably those directly comparing pSS with non‐SS sicca (Saúco et al. 2023; Sembler‐Møller et al. 2019), found no or moderate disease effect (Kim et al. 2022; Saúco et al. 2023) once hyposalivation and oral‐health covariates were accounted for (Table 2).

##### 3.3.2.3 Enriched and Decreased Taxa

pSS saliva frequently exhibited enrichment of putative pathobionts, notably *Prevotella* (Alam et al. 2020; Saúco et al. 2023; Wang et al. 2022; Xie et al. 2024; Zhou, Cai, et al. 2018) and *Veillonella* (Siddiqui et al. 2016; Wang et al. 2022; Xie et al. 2024; Zhou, Ling, et al. 2018; Table 3), and relative depletion of “health‐associated commensals” such as *Neisseria* (Alam et al. 2020; Rusthen et al. 2019; Wang et al. 2022; Zhou, Cai, et al. 2018; Zhou, Ling, et al. 2018), *Haemophilus* (Alam et al. 2020; Rusthen et al. 2019; van der Meulen et al. 2018b; Wang et al. 2022; Zhou, Ling, et al. 2018) and *Lautropia* (Alam et al. 2020; Wang et al. 2022; Zhou, Cai, et al. 2018). Some studies also reported reduced *Leptotrichia* (Alam et al. 2020; Sharma et al. 2020; Xie et al. 2024). *Streptococcus* showed inconsistent trends, being elevated in some pSS cohorts (Alam et al. 2020; Kim et al. 2022; Siddiqui et al. 2016) and reduced in others (Saúco et al. 2023; van der Meulen et al. 2018b; Zhou, Cai, et al. 2018). Species‐level differences varied across studies. In those that included non‐SS sicca controls, many of these taxonomic shifts were attenuated or lost once reduced salivary flow was accounted for, indicating that hyposalivation may represent a dominant ecological driver (Rusthen et al. 2019; Saúco et al. 2023; Sembler‐Møller et al. 2019; Wang et al. 2022). Conversely, a subset of studies that adjusted for flow or used matched sicca controls still identified pSS‐associated taxa (e.g., strong association of *Prevotella melaninogenica* with pSS; Alam et al. 2020), suggesting disease‐specific effects in a subset of patients.

#### 3.3.3 Buccal Swabs

##### 3.3.3.1 α‐Diversity

Li et al. (2016, small buccal‐swab cohort) and de Paiva et al. (2016, small dorsal‐tongue cohort) reported a reduced α‐diversity in pSS patients (but the results were not significant in Li et al. study), while van der Meulen et al. (2018a, larger buccal‐swab cohort) detected no overall α‐change between pSS, non‐SS sicca, and HCs, though trends toward reduced richness and diversity were observed in pSS (Table 2).

##### 3.3.3.2 β‐Diversity

β‐diversity generally distinguished pSS from HCs, with increased inter‐individual heterogeneity among pSS patients (de Paiva et al. 2016; van der Meulen et al. 2018a; Table 2), but not between pSS and non‐SS sicca (van der Meulen et al. 2018a). van der Meulen et al. (2018a) found that diagnostic group (pSS, non‐SS sicca, HC) and unstimulated salivary secretion rate explained a modest but comparable share of community variance, reinforcing that both autoimmunity and dryness shape the mucosal microbiome.

##### 3.3.3.3 Enriched and Decreased Taxa

Taxonomic analyses indicated a consistent pattern of dysbiosis in pSS marked by a reduction in Proteobacteria and health‐associated commensals, and a relative enrichment of Firmicutes and anaerobic taxa (Table 3). de Paiva et al. (2016) reported increased *Streptococcus* and decreased *Leptotrichia* and *Fusobacterium* in tongue swabs. In buccal swabs, Li et al. (2016) observed reduced levels of *Haemophilus*, *Neisseria*, *Comamonas*, and *Granulicatella*, alongside increased abundance of environmental or low‐abundance genera like *Leucobacter*, *Delftia*, and *Ralstonia*. van der Meulen et al. (2018a) identified a broader dysbiotic signature characterized by depletion of Proteobacteria (e.g., *Haemophilus*, *Neisseria*, and *Lautropia*) and enrichment of Firmicutes and Actinobacteria (e.g., *Lactobacillus*, *Atopobium*, *Parvimonas*, *Scardovia*, *Alloscardovia*, *Bifidobacterium*). However, many of these microbial shifts were attenuated after adjusting for saliva flow rate, indicating that hyposalivation significantly contributes to dysbiosis.

#### 3.3.4 Gingival Crevicular Fluid

In the single GCF study (Martínez‐Nava et al. 2023), conducted mainly in pSS patients with PD (Table 1), α‐diversity was higher in pSS patients versus HCs, but β−diversity was similar (Table 2). Taxonomically, pSS GCF microbiota was enriched in PD‐associated genera (*Prevotella, Fusobacterium, Veillonella, Leptotrichia, Oribacterium, Actinomyces*, *Porphyromonas*) and depleted for *Selenomonas* and members of the *Lactobacillales* order. Multivariable models (adjusting for age and salivary flow) highlighted several pSS‐associated taxa, many within *Prevotella* and *Gemella* genera (Table 3).

### 3.4 Systemic Lupus Erythematosus

#### 3.4.1 Study Characteristics

Five studies were included with a total of 314 SLE patients (Corrêa et al. 2017; Guo et al. 2023; Li et al. 2020; Liu et al. 2021; van der Meulen et al. 2019), predominantly women, from Asia, Europe, and South America. All studies compared SLE patients to HCs (except for van der Meulen et al. (2019), which compared SLE to pSS patients) (Table 1) and used 16S rRNA sequencing (Table 2). They analyzed buccal or tongue swabs (Guo et al. 2023; Li et al. 2020; van der Meulen et al. 2019), oral washings (van der Meulen et al. 2019), saliva (Liu et al. 2021), or subgingival dental plaque (Corrêa et al. 2017). Corrêa et al. (2017) conducted periodontal assessments and found a significantly higher prevalence and severity of PD in SLE patients compared to HCs (67% vs. 54%). van der Meulen et al. (2019) observed that oral dryness was less severe in SLE compared to pSS patients (Table 1).

#### 3.4.2 α‐ and β‐Diversity

α‐diversity findings were inconsistent (Table 2). In saliva/oral washings and swab samples, three studies reported significantly increased α‐diversity in SLE patients compared to HCs (Guo et al. 2023; Liu et al. 2021) or to pSS patients (van der Meulen et al. 2019). In contrast, Li et al. (2020) observed reduced α‐diversity in buccal swabs from SLE patients. Corrêa et al. (2017) found that α‐diversity patterns varied with periodontal status in SLE patients versus HCs (higher in non‐PD SLE patients; lower in SLE patients with PD).

β‐diversity consistently separated SLE patients from HCs (or pSS patients in van der Meulen et al. 2019), regardless of niche. In Corrêa et al.'s (2017) study, β‐diversity also varied depending on periodontal status. Li et al. (2020) found no significant differences in α‐ or β − diversity between new‐onset and treated SLE, or between those in active versus remission phases, suggesting that these microbial alterations may occur independently of disease activity. Notably, van der Meulen et al. (2019) showed that oral health variables (reported gum condition, tooth number, xerostomia) explained more variance in β‐diversity (10%–16%) than disease phenotype alone (8%–9%).

#### 3.4.3 Enriched and Decreased Taxa

Key genera consistently enriched in SLE included *Prevotella* (Guo et al. 2023; Liu et al. 2021; van der Meulen et al. 2019), *Veillonella* (Guo et al. 2023; Li et al. 2020; Liu et al. 2021), and *Selenomonas* (Guo et al. 2023; Liu et al. 2021), indicating a shift toward pro‐inflammatory anaerobic‐dominated communities. In contrast, commensal genera such as *Streptococcus* (Guo et al. 2023; Liu et al. 2021), *Rothia* (Guo et al. 2023; Li et al. 2020; Liu et al. 2021), and *Granulicatella* (Guo et al. 2023; Liu et al. 2021) were frequently depleted in SLE. These dysbiotic patterns were observed across different oral niches (Table 3). Interestingly, Li et al. (2020) detected similar alterations in newly diagnosed, treatment‐naïve patients, suggesting that dysbiosis may precede therapy and could potentially contribute to disease onset or progression, rather than being a secondary effect of immunosuppressive medications.

When comparing SLE to pSS patients, van der Meulen et al. (2019) found that *Prevotella* and *Granulicatella* were both enriched in SLE, while *Lactobacillus* was relatively depleted. These findings suggest that although both systemic AID share features of oral dysbiosis, SLE is characterized by a distinct microbial profile. 

Only three studies (Corrêa et al. 2017; Li et al. 2020; Liu et al. 2021) reported species‐level microbiota differences between SLE and HCs. Corrêa et al. (2017) stratified their subgingival plaque analysis by periodontal status and identified several anaerobic species (e.g., *Prevotella nigrescens* , *Selenomonas noxia* ) as significantly enriched in SLE patients, even in periodontally healthy sites. These findings suggest the existence of a disease‐specific oral dysbiosis in SLE, distinct from the microbial changes typically associated with PD alone.

### 3.5 Anti‐Neutrophil Cytoplasmic Antibody–Associated Vasculitis

A single study in acute AAV (*n* = 25; Esberg et al. 2022) (Table 1) reported markedly reduced α‐diversity, a contracted core microbiome, and high inter‐individual variability (Table 2). Taxonomically, AAV saliva was enriched for genera such as *Lactobacillus, Staphylococcus, Arthrospira, Ruminococcaceae G‐1, and Cardiobacterium*. In contrast, several genera including *Haemophilus, Fusobacterium, Alloprevotella, Leptotrichia*, and *Schaalia* were significantly depleted (Table 3). Although recent antibiotic exposure was common (major confounder), a similar pattern, with an enrichment of several PD‐associated species (e.g., *Aa*, multiple *Actinomyces* spp., *Fusobacterium* spp., *Dialister invisus, Prevotella nigrescens, Tannerella forsythia* and five other *Prevotella* spp.), persisted in antibiotic‐naïve patients (Table 3).

## 4 Discussion

**FIGURE 2:**
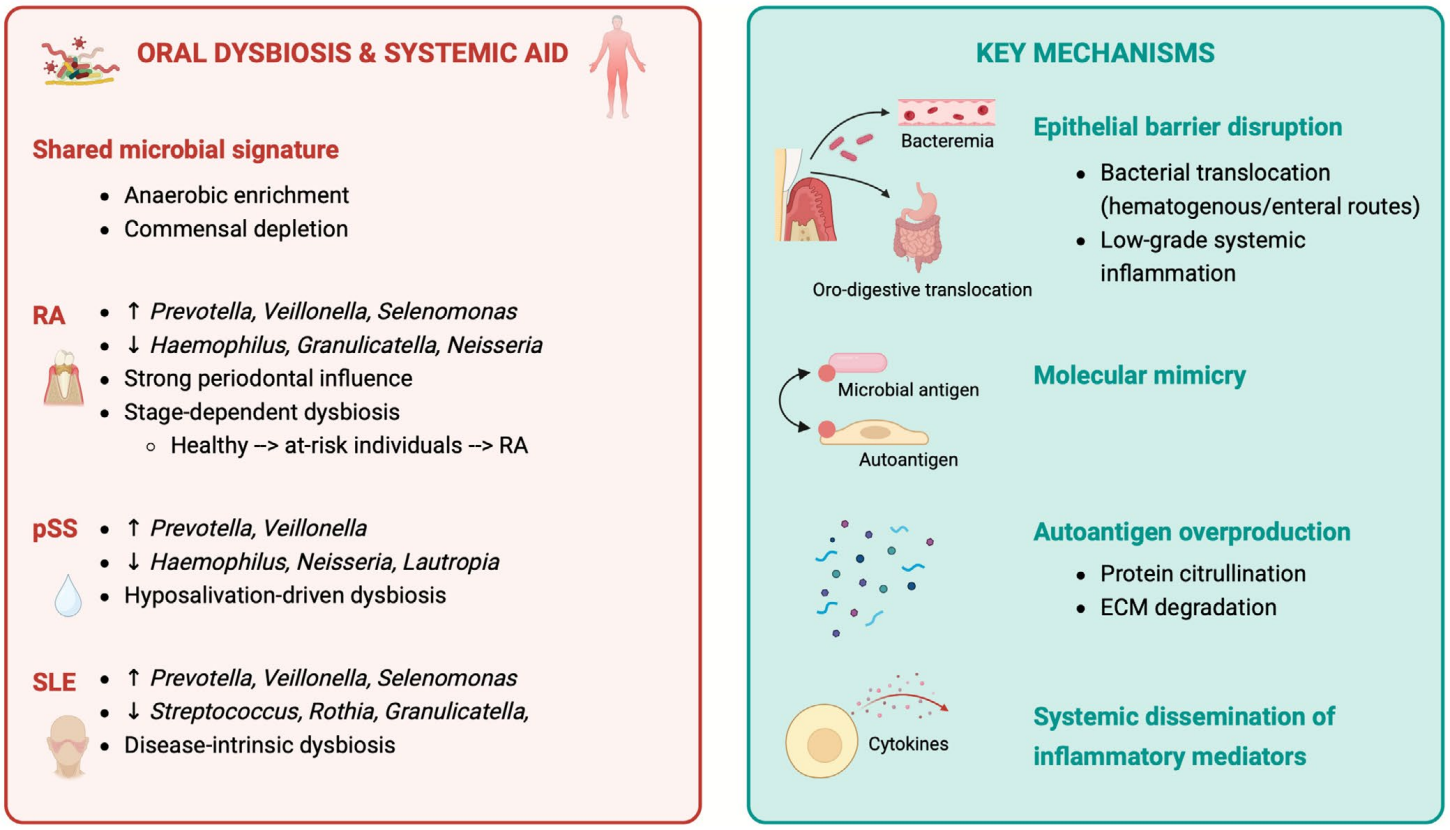
Oral dysbiosis in systemic autoimmune diseases. Systemic autoimmune diseases display shared and disease‐specific oral microbiome alterations. Genus‐level microbial signatures and key mechanisms linking oral dysbiosis to systemic immune activation are summarized. Created in BioRender. https://BioRender.com/3as1ez6. AID, autoimmune disease; ECM, extracellular matrix; pSS, primary Sjögren's syndrome; RA, rheumatoid arthritis; SLE, systemic lupus erythematosus.

This systematic review synthesizes findings from 42 observational studies including over 2100 patients with systemic AID. Despite methodological heterogeneity, a shared dysbiotic pattern emerged across RA, pSS, SLE, and AAV (Figure 2). Consistent with earlier big‐data overviews (Gao et al. 2022), our review extends these findings by integrating more recent studies and additional AID, including AAV, further delineating disease‐specific microbial signatures.

### 4.1 Disease‐Specific Insights

#### 4.1.1 Rheumatoid Arthritis

Different oral habitats, in particular subgingival plaque, displayed enrichment of anaerobic and pro‐inflammatory taxa and depletion of commensals (Figure 2). However, many differences disappeared once periodontal status was controlled for, suggesting that the local inflammation strongly influences periodontal microbiota composition. The detection of *Pg* in at‐risk individuals (Cheng et al. 2021) supports its proposed pathogenic role in early autoimmunity via protein citrullination and ACPA generation (Wegner et al. 2010), although this finding, reported in a single study, requires confirmation in independent cohorts. In contrast, salivary microbiota alterations were consistently observed across studies, even in individuals at risk of developing RA, suggesting that they may better capture systemic immune dysregulation than plaque‐based profiles. The progressive compositional shift from HCs to at‐risk, and then to RA patients (Tong et al. 2019), further supports the notion that dysbiosis may precede clinical disease onset. Interestingly, the single study investigating the oral virome (Guo et al. 2022) revealed that its composition was also influenced by immunosuppressive treatments.

#### 4.1.2 Primary Sjögren's Syndrome

In pSS, most studies showed that reduced salivary flow was a dominant ecological driver, shifting the salivary microbiota from Proteobacteria toward anaerobic Firmicutes. Nevertheless, several taxa remained altered after adjustment for salivary flow or when compared with non‐SS sicca controls, suggesting additional disease‐specific effects. In the saliva and oral washings, the consistent enrichment of *Prevotella* and *Veillonella* and depletion of health‐associated commensals such as *Neisseria* and *Haemophilus* mirror the dysbiosis observed in RA, suggesting common mucosal immune mechanisms. The single GCF‐based study, conducted mainly in pSS patients with PD, showed enrichment of genera commonly linked to PD, indicating that local periodontal inflammation may amplify systemic immune‐microbial interactions. Overall, pSS‐associated dysbiosis reflects both ecological shifts associated with reduced salivary secretion and disease‐specific immune alterations.

#### 4.1.3 Systemic Lupus Erythematosus

In SLE, all studies demonstrated altered β‐diversity and a consistent enrichment of anaerobic genera (*Prevotella*, *Veillonella*, *Selenomonas*) with depletion of commensals (*Streptococcus*, *Rothia*, *Granulicatella*). These microbial changes appeared independent of disease activity, treatment or oral niche, suggesting a stable disease‐intrinsic dysbiosis. In subgingival plaque, several anaerobic species remained enriched, even in periodontally healthy SLE patients, indicating that immune dysfunction itself may drive microbial selection. Comparisons with pSS patients revealed partially overlapping yet distinct microbial communities, suggesting disease‐specific selection pressures (van der Meulen et al. 2019).

#### 4.1.4 ANCA‐Associated Vasculitis

Evidence for AAV remained limited to a single study (Esberg et al. 2022) that showed severe salivary dysbiosis characterized by reduced core diversity and enrichment of periodontopathogenic species. Although antibiotic use was frequent, similar alterations persisted in antibiotic‐naïve patients, indicating severe ecological disruption during active disease.

Data for other systemic AID such as SSc, APL, MCTD, myositis, and systemic vasculitides other than AAV were lacking, representing important research gaps.

### 4.2 Potential Mechanisms Linking Oral Dysbiosis to Systemic Immune Activation

Oral dysbiosis has emerged as a critical driver of systemic autoimmunity through several interrelated mechanisms. In conditions such as PD, disruption of the host–microbe equilibrium triggers local chronic inflammation, leading to bacterial translocation. Oral pathogens and their products, but also orally primed inflammatory cells, can migrate through ulcerated gingival epithelium into the bloodstream, inducing chronic low‐grade systemic inflammation (Hajishengallis and Chavakis 2021; Huang et al. 2023; Kitamoto et al. 2020; Spivak et al. 2022; Suárez et al. 2020; Vieira et al. 2018). This process may contribute to both the initiation and amplification of AID. In addition, several taxa can resist gastric acidity and colonize the intestine, promoting gut dysbiosis, barrier dysfunction, and mucosal immune activation (Kinashi and Hase 2021) and supports the concept of “oral–gut immune axis”, linking oral dysbiosis to intestinal and systemic immune dysregulation (Kunath et al. 2024). Oral bacteria can also trigger molecular mimicry, where microbial antigens share epitopes with host proteins, leading to the production of cross‐reactive antibodies (Greiling et al. 2018; Suárez et al. 2020). Additionally, bacterial enzymes can drive autoantigen overproduction through protein citrullination (PPAD from *Pg*) and extracellular matrix degradation (e.g., gingipains), enhancing autoreactive responses (Suárez et al. 2020; Wegner et al. 2010). Finally, cytokine release, particularly IL‐1β, IL‐6, and IL‐17, may further amplify autoimmunity by promoting Th17 differentiation and sustained immune activation (Huang et al. 2023; Kitamoto et al. 2020).

### 4.3 Limitations

Interpretation of the present findings must consider several limitations, particularly the lack of methodological standardization across studies. High inter‐study variability resulted mainly from heterogeneity in sampling sites, but also from collection protocols (e.g., stimulated versus unstimulated saliva). Each oral niche represents a distinct ecological habitat. Therefore, findings from one site cannot be directly extrapolated to another. The collection timing, dietary restrictions, and oral hygiene before sampling were not always standardized, introducing additional variability. Heterogeneity in sequencing platforms, reference databases, analytical pipelines, and diversity metrics further hampered quantitative comparison. Most studies relied on 16S rRNA gene sequencing, which limits species‐level and functional interpretation. Shotgun metagenomics, used in only three studies (Cheng et al. 2021; Guo et al. 2022; Zhang et al. 2015), revealed additional insights into microbial metabolism, but also into the oral virome. Confounding factors, including periodontal status, salivary flow, antibiotic exposure, and smoking, were inconsistently reported or controlled. Only about one third of studies included objective periodontal evaluation, while others relied on self‐reported data. The influence of immunosuppressive therapies on the oral microbiota remained underexplored, although recent evidence (Guo et al. 2022; Wang et al. 2022; Zhang et al. 2015) suggests therapy‐specific alterations in both bacteriome and virome profiles.

Study design represented another critical limitation. The predominance of cross‐sectional studies precludes causal inference. Nevertheless, cohorts of at‐risk individuals suggest that oral dysbiosis may precede or amplify autoimmune activation. Moreover, control groups varied widely, ranging from healthy individuals to OA or non‐sicca controls, further complicating comparison across AID. We found no data for SSc, despite the marked oral involvement (Jung et al. 2023), but also for MCTD, APL, myositis, and systemic vasculitides other than AAV.

### 4.4 Translational Implications

The oral cavity is easily accessible and repeatedly sampleable, positioning the oral microbiome as a promising non‐invasive window into systemic autoimmunity. Dysbiotic patterns shared by different systemic AID raise the possibility of using oral microbial profiles as non‐invasive biomarkers for disease risk, activity, or even for therapeutic response. For instance, the progressive salivary microbiota shifts from healthy individuals to at‐risk, and ultimately to RA patients, reported by Tong et al. (2019) suggest a potential diagnostic trajectory. Moreover, the overlap between oral and systemic inflammation supports the rationale of integrating periodontal treatment alongside personalized oral‐hygiene protocols into the management of AID. Periodontal treatment has been associated with improvements in systemic inflammatory markers and disease activity in RA, and to a lesser extent in SLE (Fabbri et al. 2014; Sun et al. 2021). Nevertheless, controlled interventional trials remain limited. Modulating the oral microbiota (e.g., use of prebiotics and probiotics, antimicrobial molecules, phototherapy) may therefore represent a promising adjunctive strategy, but requires rigorous evaluation (Huang et al. 2023).

### 4.5 Future Directions

Future research should (a) adopt standardized protocols for oral sampling, periodontal assessment, and sequencing analysis to improve reproducibility; (b) include longitudinal and pre‐clinical cohorts to delineate temporal relationships between dysbiosis and disease onset; (c) expand beyond the bacteriome to include the oral mycobiome, virome, and host transcriptome or metabolome; (d) evaluate therapeutic influences by profiling the oral microbiota before and after immunomodulatory or periodontal interventions; (e) address under‐represented AID; (f) characterize the functional impact of oral dysbiosis through mechanistic studies.

## 5 Conclusion

Although methodological heterogeneity limits comparison between the studies, the collective data indicate that oral dysbiosis may actively contribute to systemic immune activation. The oral cavity thus emerges as a window into systemic autoimmunity and a potential target for immunomodulatory and microbiota‐directed interventions.

## Author Contributions

**Sophie Jung:** conceptualization, investigation, writing – original draft, formal analysis, data curation. **Eirini Militsi:** investigation, writing – original draft, formal analysis. **Olivier Huck:** conceptualization, investigation, writing – review and editing, validation, methodology, supervision.

## Funding

This work was supported by Hôpitaux Universitaires de Strasbourg (PRI 2023 ‐ HUS n°9149) and Université de Strasbourg (IDEX RECH EXPL 2024).

## Conflicts of Interest

The authors declare no conflicts of interest.

## Supporting information


**Data S1:** Supporting Information.

**Table S1:** Prisma Checklist.

## Data Availability

Data sharing not applicable to this article as no datasets were generated or analyzed during the current study.
