# Supplementary material for: Oral Microbiome in Systemic Autoimmune Diseases: A Systematic Review
Source: Oral Dis. 2026 Mar 27;32(5):1237–72. doi: 10.1111/odi.70215 (PMC13365020; doi:10.1111/odi.70215)
Supplement: Supplementary file 1 — Data S1: Supporting Information. [file ODI-32-1237-s002.docx]

**SUPPLEMENTARY METHODS**

**SEARCH EQUATIONS**

The search equations that have been used are detailed in the following table:

| **DATABASE** | **SEARCH EQUATION** |
| --- | --- |
| **PubMed/MEDLINE** | ("Autoimmune Diseases"[Mesh] OR "Lupus Erythematosus, Systemic"[Mesh] OR "Arthritis, Rheumatoid"[Mesh] OR "Scleroderma, Systemic"[Mesh] OR "Sjogren's Syndrome"[Mesh] OR "Antiphospholipid Syndrome"[Mesh] OR "Myositis"[Mesh] OR "Systemic Vasculitis"[Mesh] OR "Mixed Connective Tissue Disease"[Mesh]) AND ("mouth"[MeSH Terms] OR "mouth"[All Fields] OR "oral"[All Fields]) AND "microbiota"[MeSH Terms] |
| **Web of Science (WoS)** | ALL=(systemic autoimmune disease OR systemic lupus erythematosus OR rheumatoid arthritis OR systemic sclerosis OR systemic scleroderma OR Sjögren's syndrome OR antiphospholipid syndrome OR myositis OR systemic vasculitis OR mixed connective tissue disease) AND ALL=(oral microbiota OR periodontal microbiota OR dental plaque microbiota OR salivary microbiota OR oral microbiome OR periodontal microbiome OR dental plaque microbiome OR salivary microbiome) |

**ELIGIBILITY CRITERIA**

The following PECO framework was used to formulate the focused question (Morgan et al., 2018):

- P (population): adult patients over 18 years of age
- E (exposure): what is the effect of a systemic AID *versus*
- C (comparator): absence of systemic autoimmunity on
- O (outcomes): oral (salivary and/or periodontal/dental plaque and/or oral mucosa and/or gingival crevicular fluid [GCF]) microbiota composition and/or diversity
- S (type of studies included): observational studies

To be eligible for inclusion, the studies had to meet the following criteria:

- Original research published in peer-reviewed journals.
- Descriptive and observational human studies, e.g. cross-sectional, cohort, longitudinal and case-control studies.
- Patients with a diagnosis of systemic AID based on validated classification criteria (see below).
- Evaluation of the diversity and/or the composition of the oral microbiota (salivary and/or periodontal and/or buccal mucosa and/or GCF microbiota)

Exclusion criteria were as follows:

- Patients diagnosed with organ-specific AID, other systemic diseases or with more than one systemic AID.
- Patients with imprecise diagnosis of systemic AID (not based on validated diagnosis criteria or no clarification of the diagnosis criteria that have been used).
- No description of the oral microbiota diversity and/or composition.
- Animal or *in vitro* studies.
- Reviews, comments, editorials.
- Language other than English and French.

**CLASSIFICATION CRITERIA OF SYSTEMIC AUTOIMMUNE DISEASES**

The following validated classification criteria were used for the inclusion of patients with systemic autoimmune diseases:

| **SYSTEMIC AID** | **CLASSIFICATION CRITERIA** |
| --- | --- |
| **RA** | - 1987 American Rheumatism Association (Arnett et al., 1988) - 2010 ACR/EULAR (Aletaha et al., 2010). |
| **SS** | - 2002 American European Consensus Group (AECG) (Vitali et al., 2002) - 2012 ACR (Shiboski et al., 2012) - 2016 ACR/EULAR 2016 (Shiboski et al., 2017). |
| **SLE** | - 1982/1997 ACR (Hochberg, 1997) - 2012 Systemic Lupus International Collaborating Clinics (SLICC) (Petri et al., 2012) - 2019 ACR/EULAR (Aringer et al., 2019). |
| **SSc** | - 1980 ACR (Masi et al., 1980) - 2013 ACR/EULAR (van den Hoogen et al., 2013). |
| **APL** | - Sapporo (Miyakis et al., 2006; Wilson et al., 1999) - 2023 ACR/EULAR (Barbhaiya et al., 2023). |
| **MCTD** | - 2019 diagnostic criteria from the Japan research committee of the ministry of health, labor, and welfare for systemic AID (Tanaka et al., 2021) |
| **Systemic vasculitis** | - 1990 ACR criteria for EGPA/GPA (Leavitt et al., 1990; Masi et al., 1990) - 2007 European Medicines Agency algorithm for AAV and polyarteritis nodosa (Watts et al., 2007) - 2012 revised International Chapel Hill Consensus Conference nomenclature of vasculitides (Jennette et al., 2013) - 2022 ACR/EULAR classification criteria for EGPA/GPA (Robson et al., 2022) - 2022 ACR/EULAR classification criteria for Takayasu arteritis (Grayson et al., 2022) - 2022 ACR/EULAR classification criteria for microscopic polyangiitis (Suppiah et al., 2022). |
| **Myositis** | - 2017 EULAR/ACR (Lundberg et al., 2017). |

**Abbreviations:** AAV: anti-neutrophil cytoplasmic antibody-associated vasculitis; ACR: American College of Rheumatology; APL: primary antiphospholipid syndrome; EGPA: eosinophilic granulomatosis with polyangiitis, EULAR: European League Against Rheumatism; GPA: granulomatosis with polyangiitis; MCTD: mixed connective tissue disease; RA: rheumatoid arthritis; SLE: systemic lupus erythematosus; SS: Sjögren syndrome; SSc: systemic sclerosis

**SELECTION PROCESS**

The Rayyan web-tool was used to identify the duplicates (Ouzzani, Hammady, Fedorowicz, & Elmagarmid, 2016). A first screening of the article titles and abstracts was made by two independent reviewers (S.J. and O.H.), to eliminate irrelevant articles according to the exclusion criteria.

**SUPPLEMENTARY REFERENCE**

Ouzzani, M., Hammady, H., Fedorowicz, Z., & Elmagarmid, A. (2016). Rayyan-a web and mobile app for systematic reviews. *Systematic Reviews*, *5*(1), 210. doi: 10.1186/s13643-016-0384-4
